# Enhancing transcriptome analysis in medicinal plants: multiple unigene sets in *Astragalus membranaceus*

**DOI:** 10.3389/fpls.2024.1301526

**Published:** 2024-02-07

**Authors:** Ji-Nam Kang, Mok Hur, Chang-Kug Kim, So-Hee Yang, Si-Myung Lee

**Affiliations:** ^1^ Genomics Division, National Institute of Agricultural Sciences, Jeonju-si, Jeollabuk-do, Republic of Korea; ^2^ Department of Herbal Crop Resources, National Institute of Horticultural & Herbal Science, Eumseong-gun, Chungcheongbuk-do, Republic of Korea

**Keywords:** PacBio, rnaSPAdes, Trinity, phenylpropanoid biosynthesis, transcriptome analysis

## Abstract

*Astragalus membranaceus* is a medicinal plant mainly used in East Asia and contains abundant secondary metabolites. Despite the importance of this plant, the available genomic and genetic information is still limited. *De novo* transcriptome construction is recognized as an essential method for transcriptome research when reference genome information is incomplete. In this study, we constructed three individual transcriptome sets (unigene sets) for detailed analysis of the phenylpropanoid biosynthesis pathway, a major metabolite of *A. membranaceus*. Set-1 was a circular consensus sequence (CCS) generated using PacBio sequencing (PacBio-seq). Set-2 consisted of hybridized assembled unigenes with Illumina sequencing (Illumina-seq) reads and PacBio CCS using rnaSPAdes. Set-3 unigenes were assembled from Illumina-seq reads using the Trinity software. Construction of multiple unigene sets provides several advantages for transcriptome analysis. First, it provides an appropriate expression filtering threshold for assembly-based unigenes: a threshold transcripts per million (TPM) ≥ 5 removed more than 88% of assembly-based unigenes, which were mostly short and low-expressing unigenes. Second, assembly-based unigenes compensated for the incomplete length of PacBio CCSs: the ends of the 5`/3` untranslated regions of phenylpropanoid-related unigenes derived from set-1 were incomplete, which suggests that PacBio CCSs are unlikely to be full-length transcripts. Third, more isoform unigenes could be obtained from multiple unigene sets; isoform unigenes missing in Set-1 were detected in set-2 and set-3. Finally, gene ontology and Kyoto Encyclopedia of Genes and Genomes analyses showed that phenylpropanoid biosynthesis and carbohydrate metabolism were highly activated in *A. membranaceus* roots. Various sequencing technologies and assemblers have been developed for *de novo* transcriptome analysis. However, no technique is perfect for *de novo* transcriptome analysis, suggesting the need to construct multiple unigene sets. This method enables efficient transcript filtering and detection of longer and more diverse transcripts.

## 1 Introduction

RNA sequencing (RNA-seq) is a powerful technique for studying transcriptomes in a variety of organisms. In particular, when reference genomes are not available, *de novo* whole-transcriptome (unigene set) construction using RNA-seq is essential for transcriptome research (Clarke et al., 2013; Hölzer and Marz, 2019). RNA-seq can achieve both aspects of gene discovery and expression quantification and has become a standard technology across life science research beyond the category of genomics (Conesa et al., 2016).

Various RNA-seq techniques have been applied to organisms. Illumina sequencing (Illumina-seq) is a technology that assembles short reads into contigs. Because these contigs can represent specific transcripts and their isoforms, accurate contig assembly is considered the most important factor in transcriptome analysis using Illumina-seq (Hölzer and Marz, 2019). To date, various assemblers have been developed and evaluated, including Trinity, Oases, Trans-ABySS, SOAPdenovo-Trans, IDBA-Tran, Bridger, BinPacker, Shannon, and SPAdes (Clarke et al., 2013; Hölzer and Marz, 2019). However, although the parameter settings of each assembler and the genetic characteristics of the target organisms are key variables for contig assembly, these studies have shown that no assembler can optimally assemble Illumina-seq reads and that short sequencing reads cannot completely recover homologous transcripts without reference genome information because of the presence of complex isoforms (Li et al., 2017; Bushmanova et al., 2019; Hölzer and Marz, 2019; Prjibelski et al., 2020). The single-molecule real-time (SMRT) system of PacBio sequencing (PacBio-seq) overcomes the limitations of existing assemblers because it does not require assembly (Roberts et al., 2013; Gonzalez-Garay, 2016; Li et al., 2017). SMRT uses a pair of hairpin sequencing adapters called SMRTbells to generate circularized single-target DNA molecules (Pollard et al., 2018). The circular DNA molecule generates a kilobase-sized full-length (FL) transcript through a zero-mode waveguide (ZMW), and a circular consensus sequence (CCS) of the subreads is generated from a single ZMW. This technology can considerably increase the sensitivity of genetic isoform identification but has the disadvantages of low throughput and high error rate compared to Illumina-seq (Li et al., 2017; An et al., 2018; Pollard et al., 2018).

The optimal method for successful *de novo* transcriptome analysis is to develop a system that can resolve all the above problems. However, to date, no RNA-seq technology has overcome these problems. Therefore, researchers should consider constructing multiple unigene sets to increase the accuracy of *de novo* transcriptome analyses. The hybridization of PacBio- and Illumina-seq is the most commonly used hybrid analysis method and has been reported to show clear improvements in sequencing errors and annotation (An et al., 2018; Zhang et al., 2020). However, most of these studies focused on error correction and alternative splicing (AS) detection using RNA-seq technologies (Li et al., 2017; Weirather et al., 2017; Zhang et al., 2019; Prjibelski et al., 2020). In-depth analysis of the overall structure of the constructed transcriptomes remains poorly explored. In this study, we analyzed the overall structure of phenylpropanoid-related unigenes derived from a set of three unigenes and verified whether these unigenes exist as transcripts using reverse transcription polymerase chain reaction (RT-PCR).

**Figure 1:**
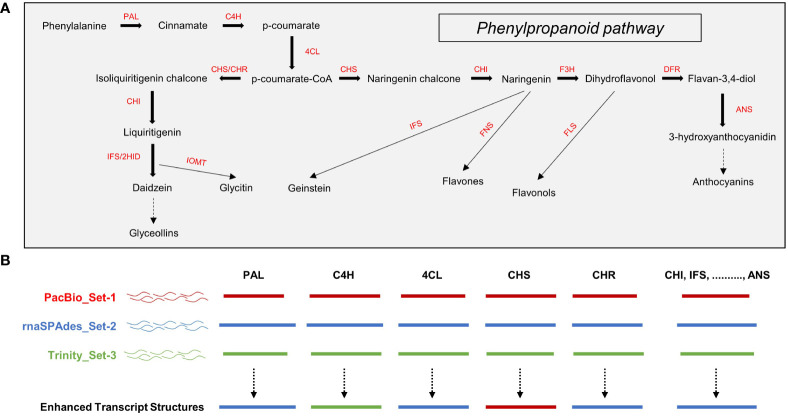
**(A)** Phenylpropanoid pathway in plants and **(B)** schematic diagram of unigene structural analysis using multiple unigene sets. PAL, phenylalanine ammonia-lyase; C4H, cinnamate 4-hydroxylase; 4CL, 4-coumaroyl-CoA ligase; CHS, chalcone synthase; CHR, chalcon reductase; CHI, chalcone isomerase; IFS, isoflavone synthase; 2HID, 2-hydroxyisoflavanone dehydratase; IOMT, isoflavone O-methyltransferase; F3H, flavonone 3-hydroxylase; FNS, flavone synthase; DFR, dihydroflavonol-4-reductase; FLS, flavonol synthase; ANS, anthocyanidin synthase.

*Astragalus membranaceus* belongs to the legume family and is primarily used as a medicinal crop in East Asia. This plant has been reported to have immunomodulatory, antioxidant, anticancer, and anti-inflammatory effects that improve human health and is rich in secondary metabolites such as phenylpropanoids and saponins (Fu et al., 2014; Chen et al., 2015). Despite the importance of this plant, knowledge of its major biosynthetic pathways and genome is still lacking, which limits its availability. In the present study, we independently constructed three unigene sets for a detailed analysis of the phenylpropanoid biosynthesis pathway (**Figure 1A**) (Yu et al., 2000; Yu and McGonigle, 2005), which is the main bioactive substance of *A. membranaceus*. Furthermore, improved transcript structures were derived by comparing the structures of key genes involved in phenylpropanoid biosynthesis from each unigene set (**Figure 1B**). Set-1 consisted of PacBio high-quality (HQ) CCSs (Gonzalez-Garay, 2016). Set-2 was constructed using rnaSPAdes, a contig assembled by integrating PacBio HQ CCS and Illumina-seq reads (Bushmanova et al., 2019). Set-3 consists of Illumina-seq contigs assembled using Trinity (Haas et al., 2013). The integrated analysis of the three unigene sets offered several advantages for profiling unigenes involved in the phenylpropanoid biosynthetic pathway compared with a single unigene set. First, it is possible to set an appropriate expression threshold through a unigene set comparison for filtering short- and low-expression assembly-based unigenes. Second, the incomplete 5`/3` untranslated region (UTR) ends of PacBio CCSs can be complemented using assembly-based unigenes. Third, more isoform unigenes could be detected than in a single unigene set. Finally, gene ontology (GO) and Kyoto Encyclopedia of Genes and Genomes (KEGG) analyses based on the differentially expressed genes (DEGs) indicated the activity of the phenylpropanoid biosynthetic pathway along with various biosynthetic pathways in roots of *A. membranaceus*. In this study, we emphasized that multiple unigene sets should be used for more accurate *de novo* transcriptome analyses in plants.

## 2 Materials and methods

### 2.1 Plant materials and isolation of total RNA

The A. *membranaceus* plants were cultivated at the National Institute of Horticulture and Herbal Science. The seeds were sown in plastic pots and grown for one month. The seedlings were then transplanted into the field and grown for four months. Leaves, stems, roots, and flowers were collected from adult plants grown for five months and immediately stored in liquid nitrogen. Three independent biological replicates were used for sampling.

Total RNA from 12 samples, including leaves, stems, roots, and flowers, was extracted using RiboExTM (GeneAll, Seoul, Korea), according to the manufacturer’s instructions. Recombinant DNase I (Takara, Shigam Japan) was used to prevent DNA contamination. RNA integrity and DNA contamination were confirmed by electrophoresis, and the RNA integrity number (RIN) was measured using a 2100 Bioanalyzer (Agilent Technologies, Santa Clara, CA, USA). All 12 samples showed RIN values above 7.0 and were used for RNA-seq (**Supplementary Figure 1**).

### 2.2 RNA sequencing and library construction

Two micrograms of RNA were used for Illumina-seq. Libraries were constructed using the TruSeq Stranded mRNA LT Sample Prep Kit (Illumina, San Diego, CA, USA). Libraries were sequenced using the Illumina HiSeq X platform (Macrogen, Seoul, Korea), and paired-end Illumina-seq data with an average length of 151 bp were generated.

PacBio-seq was performed by pooling 12 RNA samples at equal concentrations. The pooled RNA samples were then used to create libraries using the SMATer PCR cDNA Synthesis kit and DNA Template prep kit 1.0 (Pacific Biosciences, Menlo Park, CA, USA), which were sequenced using the PacBio Sequel platform (DNALink, Seoul, Korea). No library size was selected for this process.

### 2.3 Construction of unigene sets and functional annotation

All processes for PacBio-seq data analysis were performed according to the Iso-Seq3.1 pipeline in SMRTLink version 10.1 (Gordon et al., 2015). Raw subreads were generated as consensus sequences, and the primers were removed and demultiplexed. Then, all FL reads derived from the same isoform were clustered and consensus sequences were refined using subreads. CCSs with an estimated accuracy of 99% using the default parameters were classified as HQ CCSs and constructed as a unigene set-1.

Unigene set-2 was constructed by integrating the reads generated from Illumina and PacBio sequencing. Low-quality read and adapter sequences in the Illumina-seq data were removed using the Trimmomatic program (ver. 0.3.9, default parameters) (Bolger et al., 2014). An additional decontamination process to remove bacterial sequences was performed using BBduk (ver. 38.87, K-mer 31 parameters) (Bushnell, 2014). Clean Illumina-seq reads were integrated with HQ CCSs generated by PacBio-seq and assembled using rnaSPAdes assembler (ver. 3.15.0, default parameters) (Bushmanova et al., 2019). Unigene set-3 was assembled using Trinity assembler (ver. 2.12.0, default parameters), using clean Illumina-seq reads (Haas et al., 2013). Finally, sequences with > 90% homology were removed using the CD-HIT-EST program (ver.4.8.1, default parameters) (Li and Godzik, 2006). The coding regions (CDS) and protein sequences were predicted from the unigene sets using TransDecoder (ver. 5.5; default parameters) (http://transdecoder.github. io, accessed June 15, 2022).

Functional annotation of the unigenes was performed using three databases. Similarity analysis with the NCBI non-redundant protein database (DB) was performed using the BLASTX method with the DIAMOND program (ver. 0.9.30.131, cutoff e-value 1e-5) (Buchfink et al., 2015). InterProScan (ver. 5.34-73.0, default parameters) was used to search for the conserved domains of unigenes (Quevillon et al., 2005). Similarity with the Araport11 DB (https://www.arabidopsis.org/index.jsp, accessed on June 15, 2022) was analyzed using the BLASTX method (cutoff e-value 1e-5).

### 2.4 Expression profiling of unigenes

The clean Illumina-seq reads were mapped to each of the three unigene sets using Bowtie2 (ver. 2.3.5, default parameters) (Langmead and Salzberg, 2012). The mapping number of the Illumina-seq reads was measured using RNA-Seq by Expectation Maximization (RSEM, ver. 1.3.3, default parameters) method (Li and Dewey, 2011). The transcripts per million (TPM) values of the unigenes were used for expression profiling. The TPM values of unigenes related to the phenylpropanoid biosynthesis pathway were converted into Z-scores using the pheatmap package in the R studio program (Cheadle et al., 2003) and Mcquitty distance, and complete linkage were used as similarity measures to visualize the expression of unigenes.

### 2.5 Primer design and RT-PCR

The sequences of the unigenes involved in the phenylpropanoid biosynthesis pathway were aligned using the Qiagen CLC Genomics Workbench (ver. 6.0.1), which were used for the specific primer design. For RT-PCR, cDNAs were synthesized using the cDNA EcoDry Premix kit (TaKara, Kyoto, Japan) from 1 µg of total RNA. The synthesized cDNA samples were pooled and subjected to RT-PCR. The reaction mixture contained 1 µL of cDNA, 1 µL (10 pmol) of primer set, 4 µL of dNTP mixture (2.5 mM each), 4 µL of 5 × PrimeSTAR GXL buffer, and 1 µL (1.25 unit) of PrimeSTAR GXL DNA Polymerase (Takara, Kusatsu, Japan). PCR reaction was performed using an ABI GeneAmp 9700 PCR Thermal Cycler (Applied Biosystems, Waltham, USA) under the following conditions:3 min at 98°C for initial denaturation, followed by 35 cycles of 98°C for 10 s, 58°C for 15 s, and 68°C for 1 min. Amplification products were confirmed using 1% agarose gel electrophoresis. RT-PCR for amplification of individual unigenes was performed at least three times for experimental repeat.

### 2.6 Analysis of DEGs, GO terms, and KEGG pathways

Analysis of DEGs between tissue samples of *A. membranaceus* was performed using the DESeq2 package (ver. 1.28.1, accessed on 23 December 2023) in R program, and statistical significance was verified by nbinomWaldTest (Love et al., 2014). Based on an adjusted p-value of less than 0.05, unigenes showing log2 fold change > 0.5 between each sample were considered DEGs.

Blast2GO (ver. 5.2.5, accessed on 24 December 2023) was used to assign GO terms to DEGs based on the similarity analysis results (Conesa et al., 2005). KEGG Automatic Annotation Server (https://www.genome.jp/tools/kaas/, accessed on 26 December 2023) was used to analyze the KEGG pathway of DEGs with the single-directional best-hit method (Moriya et al., 2007). DEGs assigned to GO and KEGG were visualized using the R program (cutoff: basemean > 50, log2FC ≥ 2, number of enriched unigenes ≥ 3, and top 20 terms).

## 3 Results

### 3.1 RNA-seq and construction of multiple unigene sets

Illumina- and PacBio-seq were employed for transcriptome analysis of *A. membranaceus*. An average of 39,374,868 (ranging from 33,942,822 to 45,514,562) reads were generated from 12 tissues, including leaves, flowers, roots, and stems, using Illumina-seq. An average Q30 value of 94.6% (ranging from 93.9 to 94.8) ensures the quality of the reads. After trimming the contaminated sequences, an average of 36,183,471 clean reads (ranging from 31,377,018 to 41,924,484) were obtained using Illumina-seq (**Supplementary Table 1**).

PacBio-seq was performed by pooling 12 tissue RNA samples at equal concentrations. In total, 90,544 CCSs were generated from 45,497,196,751 polymerase reads. Among them, the HQ and low-quality CCS were 90,374 and 170, respectively (**Supplementary Table 2**). Clean Illumina-seq reads and PacBio HQ CCSs were used to construct multiple unigenes.

Set-1 was constructed using 90,374 PacBio HQ CCS. Set-2 comprised 291,818 contigs generated by the integrated assembly of PacBio HQ CCS and clean Illumina reads assembled using rnaSPAdes. Set-3 was assembled from Illumina clean reads using Trinity and consisted of 473,469 contigs. Finally, 32,608, 273,991, and 400,147 unigenes were selected from set-1, set-2, and set-3 after applying CD-HIT-EST (90%), respectively. Statistical information for the three unigene sets is presented in **Table 1**.

**Table 1:** Statistical analysis of the three unigene sets generated from *A. membranaceus*.

	Total unigenes			cd-hit-est (90%)			Predicted coding region		
	Set-1^*^	Set-2^*^	Set-3^*^	Set-1	Set-2	Set-3	Set-1	Set-2	Set-3
Contig number	90,374	291,818	473,469	32,608	273,991	400,147	29,944	129,808	163,658
Max length (bp)	11,361	19,621	15,943	11,361	19,621	15,943	7,893	16,197	15,297
Average length (bp)	2,234	1,180	977	2,283	1,116	926	1,248	828	787
N50 (bp)	2,413	2,441	1,845	2,484	2,348	1,754	1,515	1,095	996
GC Ratio (%)	40.73	40.69	40.48	39.99	40.79	40.59	42.53	44.98	44.96
BUSCO	83	98.9	97.1	82.8	98.7	97.1	76.7	88.5	89.6

^*^Set-1, PacBio HQ CCS; Set-2, rnaSPAdes-assembled contigs; Set-3, Trinity-assembled contigs.

### 3.2 Functional annotation and mapping of constructed unigenes

The functions of the unigenes were predicted using NCBI nr proteins, Araport11, and InterProScan DBs. Accordingly, 31,588 unigenes in set-1, 193,587 unigenes in set-2, and 256,570 unigenes in set-3 were hit in the three DBs, accounting for 96.9%, 70.7%, and 64.1% of the total unigenes, respectively (**Table 2**).

**Table 2:** Annotation of unigenes using databases.

Database	Set-1^*^		Set-2^*^		Set-3^*^	
	Number of unigenes	%	Number of unigenes	%	Number. of unigenes	%
NCBI nr proteins	31,453	96.46%	192,396	70.2%	252,891	63.20%
Araport11	16,063	49.26%	104,444	38.1%	107,868	26.96%
InterProScan	25,197	77.27%	90,705	33.1%	112,529	28.12%
Hit number	31,588	96.9%	193,587	70.7%	256,570	64.1%

^*^Set-1, PacBio HQ CCS; Set-2, rnaSPAdes-assembled contigs; Set-3, Trinity-assembled contigs.

Clean Illumina-seq reads generated from 12 tissues were individually mapped to the three unigene sets to calculate the expression values of the unigenes. These reads mapped to set-1, set-2, and set-3 with an average of 69.8% (ranging from 62.9 to 73.9), 76.8% (ranging from 72.1 to 81.8), and 75.8% (ranging from 72.2 to 80.6), respectively (**Supplementary Table 3**). The expression values of the unigenes normalized using TPM are shown in **Supplementary Tables S4–6** along with their annotation information.

### 3.3 Filtering of unigenes based on TPM value

**Figure 2:**
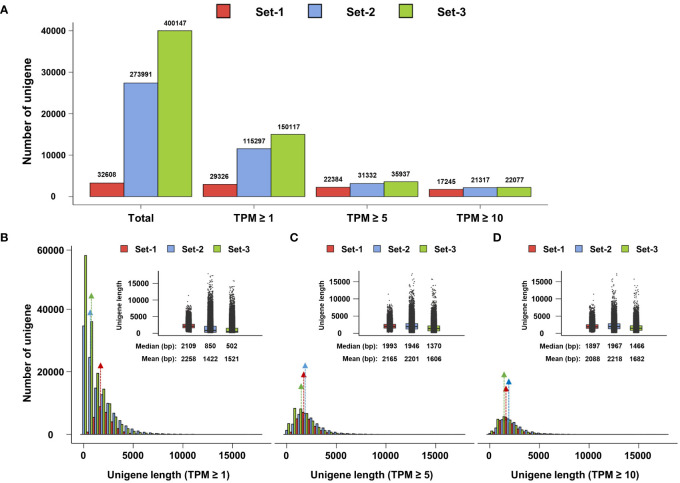
Whole unigenes filtering based on transcripts per million (TPM). **(A)** TPM threshold and unigenes number. **(B–D)** Length distribution plot of unigenes at TPM threshold. Median and mean values of unigenes at TPM thresholds were determined using box plots. Colored arrows indicate the median positions of the unigenes. Set-1, PacBio HQ CCS; Set-2, rnaSPAdes-assembled contigs; Set-3, Trinity-assembled contigs.

Low transcript expression may be the result of experimental and biological noise (Hart et al., 2013; Sha et al., 2015). Therefore, low-expression transcripts should be appropriately filtered according to the purpose of transcriptome study. To determine an appropriate TPM threshold for *de novo* transcriptome analysis of A. *membranaceus*, we investigated the effect of TPM threshold settings on unigene counts. The numbers of unigenes in Set-1, Set-2, and Set-3 were 32,608, 273,991, and 400,147, respectively. The number of set-1 unigenes reduced to 29,326, 22,384, and 17,245 at TPM ≥ 1, TPM ≥ 5, and TPM ≥ 10, respectively. The number of set-2 unigenes decreased to 115,297, 31,332, and 21,317 at TPM ≥ 1, TPM ≥ 5, and TPM ≥ 10, respectively. The number of set-3 unigenes declined to 150,117, 35,937, and 22,077 at TPM ≥ 1, TPM ≥ 5, and TPM ≥ 10, respectively. Interestingly, TPM ≥ 5 filters more than 31.3%, 88.5%, and 91% of the total unigenes in set-1, set-2, and set-3, respectively, and the number of unigenes in the three unigene sets closely matched (**Figure 2A**).

Furthermore, we examined the length distribution of unigenes remaining at each TPM threshold. At TPM ≥ 1, the median and mean of unigenes length in set-1 were similar at 2,109 bp and 2,258 bp, respectively, showing a normal distribution pattern, which means that the lengths of most unigenes in set-1 were distributed near the mean and median. The length statistics of set-1 unigenes did not differ remarkably according to the TPM threshold (**Figures 2B–D**).

On the other hand, the median and mean of set-2 unigenes at a length of TPM ≥ 1 were 850 bp and 1,422 bp, respectively, and the majority of unigenes were shorter than the median (**Figure 2B**). However, the median and mean dramatically increased to 1,946 bp and 2,201 bp, respectively, at TPM ≥ 5, and the length distribution also became similar to set-1 (**Figure 2C**). At TPM ≥ 10, the length distribution of set-2 unigenes became more similar to set-1, and the median and mean lengths were higher than those of set-1 (**Figure 2D**). Similar results were obtained for the TPM threshold of set-3 (**Figures 2B–D**).

These results indicated that the majority of the unigenes comprising set-2 and set-3, assembly-based unigenes, were short and low-expression unigenes below 5 TPM. However, at TPM ≥ 5, the fact that the length distribution of the assembly-based unigenes is similar to that of set-1 suggests that these unigenes have assembled to the appropriate length because one unigene comprising PacBio HQ CCS in set-1 would ideally represent one complete unigene.

### 3.4 Analysis of phenylpropanoid biosynthesis pathway

**Figure 3:**
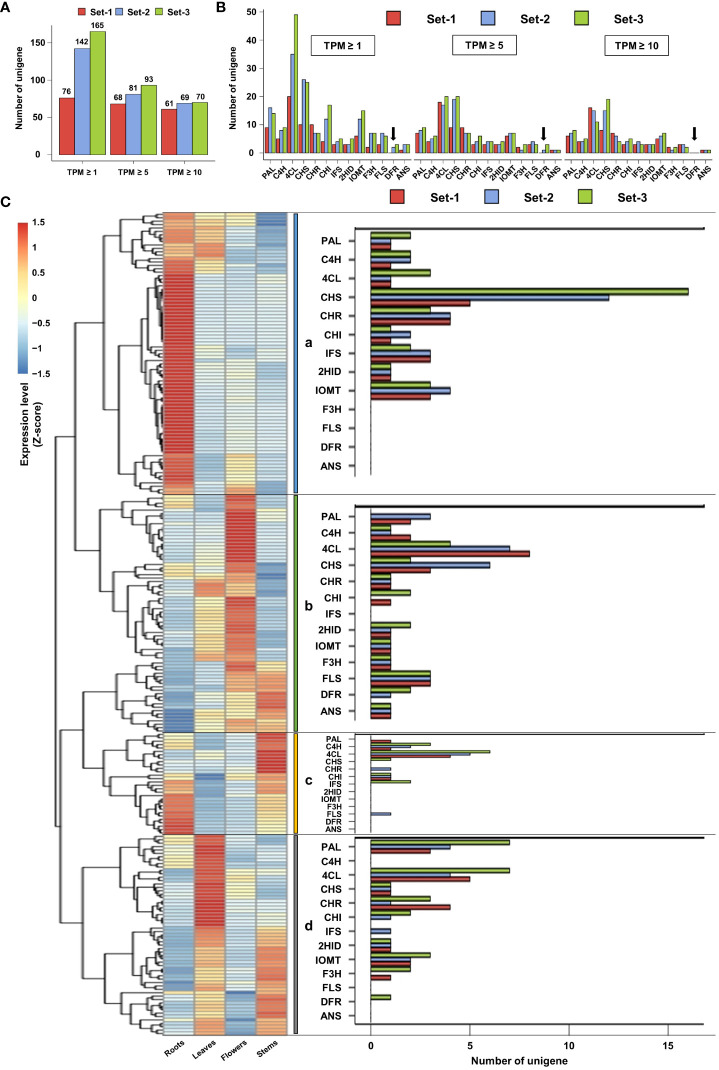
Filtering and expression analysis of unigenes involved in the phenylpropanoid pathway. **(A, B)** Filtering of unigenes involved in the phenylpropanoid pathway based on TPM. Black arrows indicate missing DFR-encoded ungenes according to the TPM threshold. **(C)** Expression analysis of phenylpropanoid-related unigenes using heatmap (TPM ≥ 5). These ungenes were divided into four groups according to their expression patterns (a–d). Set-1, PacBio HQ CCS; Set-2, rnaSPAdes-assembled contigs; Set-3, Trinity-assembled contigs.

The same TPM filtering method was applied to unigenes involved in the phenylpropanoid biosynthesis pathway. At TPM ≥ 1, 76, 142, and 165 unigenes involved in phenylpropanoid biosynthesis were identified in set-1, set-2, and set-3, respectively. The number of these unigenes was reduced to 68, 81, and 93 at a TPM threshold of ≥ 5, resulting in a removal of 10.5%, 43%, and 43.6%, respectively, compared to unigenes meeting the TPM threshold of ≥ 1. At TPM ≥ 10, the number of unigenes related to phenylpropanoid biosynthesis remained 61, 69, and 70 in set-1, set-2, and set-3, respectively, and 19.7%, 51.4%, and 57.6% of unigenes in TPM ≥ 1 were filtered (**Figure 3A** and **Supplementary Tables 7–9**). Similar to the previous results, TPM ≥ 5 closely matched the number of phenylpropanoid biosynthesis-related unigenes derived from the three unigene sets. However, missing DFR encoding unigenes were observed when examining the individual unigenes encoding key enzymes of this pathway. DFR-encoding unigenes were not detected in set-1, irrespective of the TPM threshold. DFR encoding unigenes derived from set-2 and set-3 were filtered at TPM ≥ 10 (**Figure 3B**). These results showed that key unigenes in the phenylpropanoid biosynthesis pathway may be missing in set-1 and TPM ≥ 10 threshold in set-2 and set-3.

Based on these results, we determined that TPM ≥ 5 is an appropriate TPM threshold for the analysis of the phenylpropanoid biosynthesis pathway in this study. Thus, expression analysis was performed for a total of 242 phenylpropanoid biosynthesis-related unigenes with TPM ≥ 5 from three unigene sets. Heatmap analysis revealed that the expression of these unigenes could be divided into four groups (**Figure 3C**). Group-a was mainly highly expressed in the roots and leaves (**Figure 3C**, group-a), including the IFS, 2HID, and IOMT-encoding unigenes involved in isoflavone biosynthesis (**Figure 1**). Group-b mainly showed high expression in the leaves and flowers (**Figure 3C**, group-b), which contained unigenes encoding F3H, FLS, DFR, and ANS, which are involved in flavonol and anthocyanin biosynthesis (**Figure 1**). Although unigenes were also assigned to group-c and group-d, their expression patterns and omission of key unigenes in the phenylpropanoid pathway suggested that these unigenes may be involved in other biological functions (**Figure 3C**, group-c and group-d). The expression profiles and clustering results of the 242 unigenes are shown in **Supplementary Table 10**.

### 3.5 Structural analysis of unigenes involved in phenylpropanoid biosynthesis

Because the unigenes involved in phenylpropanoid biosynthesis were derived independently from the three unigene sets, they are likely identical unigenes encoding a single enzyme. Therefore, we performed a structural analysis of the unigenes predicted to encode the same enzyme, which could provide accurate information about the single unigene structure.

We extracted the sequences of the 242 unigenes used in the expression analysis (**Supplementary Table 11**). Next, unigenes predicted to encode the same enzyme were grouped by sequence homology analysis using an alignment. One unigene derived from set-1 (Am_HQ_) was considered a single FL transcript and was assigned a unique color. Among the unigenes derived from set-2 (Am_Rn_) and set-3 (Am_Tr_), regions longer than 100 bp, corresponding to the unigenes derived from set-1, were given the same color as that of set-1. Specific primers were designed based on the structures of the aligned unigenes, and RT-PCR was used to confirm the presence of these unigene structures (**Supplementary Table 12** and **Supplementary Figure 2**). Finally, homologous unigenes expected to encode the same enzyme were rearranged, and a structural schematic diagram was constructed based on the amplified unigenes (**Supplementary Figure 2**). In this process, the unigenes for which the CDS could not be predicted were excluded.

**Figure 4:**
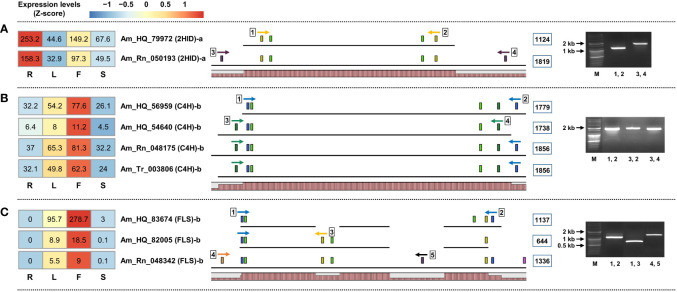
Structural schematic diagram of unigenes involved in the phenylpropanoid biosynthetic pathway derived from three unigene sets. **(A, B)** Schematic diagram of the incomplete UTR terminus of unigene derived from set-1. **(C)** Different isoforms of FLS-encoded unigene detected in set-2. The range within the light green box represents the predicted cds region. Colored boxes indicate specific primer positions. The numbers in the blue line box are the amplification sizes of the unigenes. Homology graphs are displayed at the bottom of the schematic diagram using a bar plot (right red bars).

Interesting results were obtained from structural analysis of the unigenes. First, the unigenes derived from set-1 are unlikely to be complete FL transcripts. The two unigenes encoding 2HID, Am_HQ_79972 and Am_Rn_050193, were believed to be identical unigenes encoding the same 2HID enzyme. Am_HQ_79972 unigene derived from set-1 is a typical transcript structure of a total length of 1,344 bp containing CDS and 5’/3’ UTR region. However, the 5`/3` UTR end region missing in this unigene was identified in the Am_Rn_050193 unigene derived from set-2 and was indeed an amplifiable region by RT-PCR (**Figure 4A**). This phenomenon was also confirmed for unigenes belonging to set-1. Am_HQ_56959 and Am_HQ_54640 unigenes derived from Set-1 are believed to be the same unigenes or isoforms that encode C4H. However, these two unigenes were each missing the 5’/3’ UTR end regions. RT-PCR results of the Am_Rn_048175 unigene derived from set-2 demonstrate that the Am_HQ_56959 and Am_HQ_54640 unigenes originally possess identical 5’/3’ UTR ends: Am_Rn_048175 unigene was composed of a concatenation of the Am_HQ_56959 and Am_HQ_54640 unigenes (**Figure 4B**). Missing 5`/3` UTR ends of unigenes were also observed in other phenylpropanoid biosynthesis-related unigenes derived from set-1 (**Supplementary Figure 3**).

Second, it was possible to detect additional isoform unigenes by constructing multiple unigene sets. Am_HQ_83674, Am_HQ_82005, and Am_Rn_048342 are believed to be AS forms of unigenes encoding the same FLS enzyme. Among them, Am_Rn_048342 unigenes were detected only in set-3, which was an amplifiable transcript (**Figure 4C**). Similar results were also confirmed among C4H, 4CL, CHS, and IOMT-encoding unigenes (**Supplementary Figure 4**), and these isoforms were missing from set-1 regardless of TPM filtering (**Supplementary Tables 7–9**). Finally, direct sequencing was performed to confirm whether the amplified bands matched the RNA-seq data. The amplified bands contained regions of interest consistent with the RNA-seq data (**Supplementary Figure 5** and **Supplementary Table 13**).

### 3.6 Statistical analysis of RT-PCR amplified unigenes

The main statistical data for phenylpropanoid-related unigenes amplified by RT-PCR are summarized in **Table 3**. The amplification rates of the phenylpropanoid-related unigenes derived from set-1, set-2, and set-3 were 98.5%, 67.9%, and 58.9%, respectively (**Table 3**). This suggests that the structures of the unigenes derived from set-1 were mostly substantial transcript structures, whereas set-2 and set-3 contained many misassembled unigenes. Most of the unigenes that failed RT-PCR amplification in set-2 and set-3 were caused by chimeric assembly with a mixture of their homologous unigene sequences (**Supplementary Figure 2**). However, well-constructed unigenes from set-2 and set-3 may contain unigenes missing from set-1. Sixteen isoform unigenes were detected only in set-2 and set-3. Additionally, 8, 14, and 18 unigenes encoding key enzymes involved in the phenylpropanoid biosynthesis pathway were missing in set-1, set-2, and set-3, respectively (**Table 3**). These results imply that the construction of multiple unigene sets can rescue the key unigenes involved in the biosynthetic pathway.

**Table 3:** Summary of main statistics for RT-PCR amplified unigenes.

Statistics	Set-1^*^	Set-2^*^	Set-3^*^
Number of unigenes	68	78	73
Number of unigenes (amplified)	67	53	43
Number of unigenes (failed to amplify)	1	25	30
Amplification ratio	98.5	67.9	58.9
Isoform detection	10	10	6
Omission of key enzyme encoding unigene	8	14	18

^*^Set-1, PacBio HQ CCS; Set-2, rnaSPAdes-assembled contigs; Set-3, Trinity-assembled contigs.

**Figure 5:**
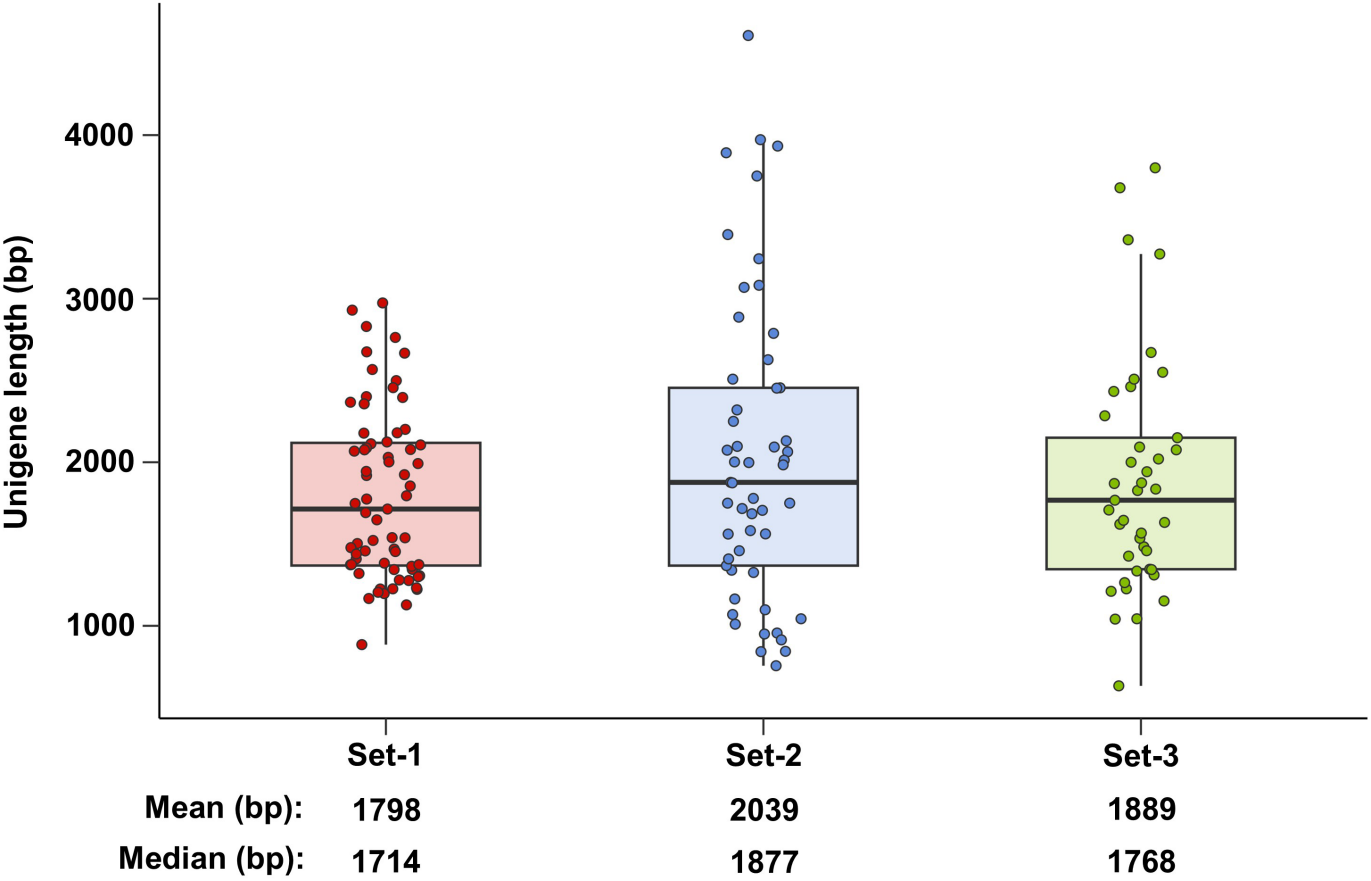
Length of phenylpropanoid-related unigenes amplified using RT-PCR. Set-1, PacBio HQ CCS; Set-2, rnaSPAdes-assembled contigs; Set-3, Trinity-assembled contigs.

A comparison of the lengths of the actual amplifiable unigenes yielded interesting results. The mean and median lengths of the phenylpropanoid-related unigenes derived from set-1 were 1,798 and 1,714 bp, respectively (**Figure 5**). However, these statistics were longer for the unigenes derived from set-2 and set-3. This is probably because well-constructed assembly-based unigenes have longer 5’/3’ UTR ends than unigenes derived from set-1. In summary, construction of multiple unigene sets has the advantage of detecting longer and more diverse unigenes.

### 3.7 Analysis of GO and KEGG enrichment based on DEGs

The previous results showed that constructing multiple unigene sets and filtering with TPM greater than 5 was an improved approach for *De novo* transcriptome analysis. Therefore, we identified the major biosynthetic pathways in *A. membranaceus* using this improved transcriptome analysis method. Since the roots of this plant are the main medicinal part, the DEG comparison consisted of three combinations based on the roots: roots vs. leaves, roots vs. flowers, and roots vs. stems.

**Figure 6:**
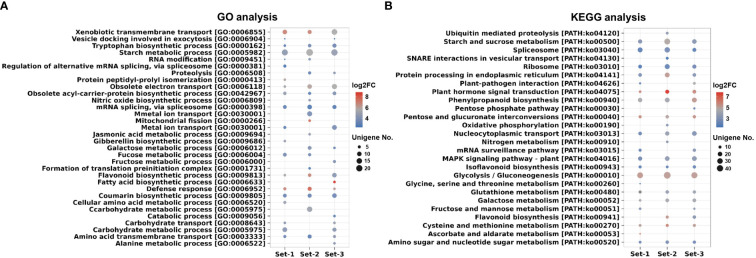
GO and KEGG analysis of filtered unigenes (TPM ≥ 5) based on DEGs (Roots vs. Leaves). **(A)** Analysis of GO terms. **(B)** Analysis of KEGG pathways. The color of the circle in dot plots represents the average log2 FC value of the unigenes assigned to each GO or KEGG. The size of the circle in dot plots indicates the number of DEGs assigned to each GO or KEGG. Set-1, PacBio HQ CCS; Set-2, rnaSPAdes-assembled contigs; Set-3, Trinity-assembled contigs. DEGs, differentially expressed genes; GO, gene ontology; KEGG, Kyoto Encyclopedia of Genes and Genomes.

In the comparison between roots and leaves, 5603, 5461, and 4771 unigenes were up-regulated in the roots, from each respective unigene set. (**Supplementary Table 14**). In GO and KEGG analysis, the DEGs were assigned to various biosynthetic pathways including flavonoid, phenylpropanoid, isoflavonoid, zeatin, diterpenoid, terpenoid backbone, ubiquinone, as well as other terpenoid-quinone and steroid pathways. In addition, metabolic pathways such as starch, fructose, carbohydrate transport, sucrose, and galactose were activated in the roots (**Figures 6A, B** and **Supplementary Tables 15, 16**). Similar results were also confirmed in other comparative combinations (**Supplementary Tables 17-20**), suggesting that the roots of *A. membranaceus* play a vital role in the synthesis of carbohydrates and pharmacological substances, potentially offering significant benefits for human health (Kang et al., 2021; Kang et al., 2022). The raw information of GO and KEGG based on DEGs was presented in **Supplementary Tables 21-23**.

## 4 Discussion

Genomic information on many organisms has been published through the development of whole-genome sequencing technologies. However, the genomes of most organisms have not been deciphered, and medicinal plants have very limited genomic information. *De novo* transcriptome analysis is recognized as an effective tool for transcriptome analysis when reference genome information is incomplete, and various tools have been developed for this purpose (Clarke et al., 2013; Haas et al., 2013; Gonzalez-Garay, 2016; Bushmanova et al., 2019). In this study, we independently constructed three unigene sets for *de novo* transcriptome analysis using PacBio sequencing, rnaSPAdes, and Trinity assembly. Although this method requires the processing of a large amount of data, we believe that it is advantageous for accurate *de novo* transcriptome analysis. The construction of multiple unigene sets revealed several interesting points.

First, a threshold TPM of ≥ 5 removed more than 88% of assembly-based unigenes. In general, transcriptome reconstruction using Illumina-seq short reads is difficult because of events such as paralog genes and AS. The vast number of contigs generated using Illumina-seq are mostly low-expressing (Hart et al., 2013) and may contain a large number of experimental false positives (Conesa et al., 2016; An et al., 2018). As a result, most of the unigenes generated from Illumina-seq may be caused by experimental or technical noise and are unlikely to be biologically active genes (Hart et al., 2013). Therefore, filtering to remove inactive transcripts is essential in Illumina-seq, and filtering low-expression transcripts is an efficient method for their identification (Hart et al., 2013; Sha et al., 2015). However, the threshold range for distinguishing low-expression transcripts varies depending on the purpose of the study, and there is no clear answer. In this study, we applied a rigorous filtering of TPM ≥ 5 for the analysis of the phenylpropanoid biosynthetic pathway. At this TPM threshold, more than 88.5% of the assembly-based unigenes were removed and most were short unigenes. Additionally, a threshold TPM ≥ 5 can almost match the unigene numbers and length of the three unigene sets (**Figure 2**). Since set-1 composed of PacBio CCSs does not require assembly and is ideally recognized as single FL transcript, the fact that unigenes number and length of set-2 and set-3 based on assembly are similar to those in set-1 implies that the remaining assembly-based unigenes in TPM ≥ 5 may have been properly constructed. Thus, the threshold TPM ≥ 5 is considered an ideal threshold to remove short and low-expresssion unigenes predicted to be inactive forms. In this study, we could not provide evidence that the unigenes removed by TPM filtering were non-functional genes. However, RNA-seq data containing incorrect information may distort future research, suggesting the need for rigorous TPM filtering for *de novo* transcriptome analysis, particularly when constructing assembly-based unigene sets using short RNA-seq reads.

Second, assembly-based unigenes can compensate for the incomplete FL of the PacBio CCS. In most unigenes involved in the phenylpropanoid biosynthesis pathway, the unigenes derived from set-2 and set-3 had extension sequences of 5’/3’ UTR end missing from the same unigene derived from set-1, and these regions were actually amplifiable (**Supplementary Figure 3**). These results suggest that the PacBio CCSs may not be complete FL transcripts. In general, the length of PacBio CCSs is dependent on the cDNA length captured by the 5’-CAP and 3’-poly A of RNA, suggesting that preparation of high purity RNA (RIN 8 or higher) sample is essential for PacBio-seq (An et al., 2018; Zhao et al., 2019). Although the 12 RNA samples used in this study were close to PacBio-seq conditions (average RIN value of 7.95), the PacBio CCSs had missing 5’/3’ UTR regions (**Supplementary Figure 3**). These results suggest that the RIN values for PacBio-seq do not ensure FL RNA and that PacBio CCSs may not be sequenced as complete FL transcripts. In this study, incomplete PacBio CCS was mainly caused by missing 5`/3` UTR end. Both ends of the RNA were relatively easily degraded, suggesting that complete FL transcripts may be difficult to derive using the PacBio-seq system, where cDNA synthesis is essential. However, amplification-based Illumina-seq is capable of high-throughput sequencing, enabling the capture of small amounts of intact FL RNA. Another reason was that the PacBio CCS reads were shortened by the consensus strategy. PacBio-seq creates a circular template by linking hairpin adapters at both ends of the target double-stranded DNA. Next, multiple subreads were generated by repetitive sequencing of the circular template using a polymerase, and the corrected CCSs were selected as the FL transcripts. It has been reported that this consensus strategy of CCS can reduce sequence errors but lower throughput and shorten the corresponding read lengths (Weirather et al., 2017; An et al., 2018). In summary, assembly-based unigenes are likely to be captured for longer than the PacBio CCS when the transcript assembly is perfect (**Figure 5**).

Third, the construction of multiple unigene sets allows the detection of more transcript isoforms. In general, PacBio-seq is superior to Illumina-seq for transcript isoform detection (Weirather et al., 2017). Transcriptome reconstruction using short reads is disadvantageous for isoform detection because of events such as (An et al., 2018). However, when short reads are integrated with long reads, such as PacBio CCS, a remarkable number of isoforms missed in the long reads are rescued (Weirather et al., 2017). This result suggests that despite the high isoform detection ability of PacBio-seq, there were clearly missed isoforms. In the present study, PacBio-seq failed to detect the isoforms of some phenylpropanoid biosynthesis-related unigenes. These isoforms were detected only in the assembly-based unigene set and most were low-expression isoforms (**Supplementary Figure 4**). These results suggest that the low throughput of PacBio-seq is not suitable for the detection of low-expression isoforms and that integration with high-throughput sequencing platforms, such as Illumina-seq, may be required.

In fact, studies utilizing hybrid assembly for transcriptome analysis have been reported in various plants. In *Cynara Cardunculus*, a hybrid assembly of Nanopore long-reads and Illumina short-reads has improved the rate of fully assembled genes and isoform detection compared to single assemblies using short-reads alone. In this process, the structure of existing genes was mostly updated through hybrid assembly, primarily involving modifications of the 5’/3’ UTR ends of the genes (Puglia et al., 2020). In *Euphorbia pulcherrima*, the unigene set constructed through a hybrid assembly of PacBio CCS and Illumina short-read increased the read mapping rate of target samples compared to single unigene set using PacBio CCS alone. These results led to successful transcriptome analysis of the phenylpropanoid pathway (Vilperte et al., 2019). Like this, hybrid assembly is recognized as an essential method for *de novo* transcriptome analysis and can produce improved transcriptome information. The construction of multiple unigene sets can provide an approach for accurate transcriptome analysis, which can be further utilized to understand the regulatory mechanisms of various biosynthesis in plants.

In conclusion, the construction of multiple unigene sets can prevent distortion in transcriptome analysis through efficient transcript filtering and enable the detection of longer and more diverse transcripts. Various platforms have been developed for *de novo* whole transcriptome construction. Advances in FL transcript detection technologies, such as PacBio-seq, have revolutionized *de novo* transcriptome analysis. However, there is still no perfect system, and this may be natural in the vast and complex world of transcriptomes. In this study, we emphasized the need to construct multiple unigene sets for more definitive transcriptome analysis.

## Data availability statement

The datasets presented in this study can be found in online repositories. The names of the repository/repositories and accession number(s) can be found below: https://nabic.rda.go.kr/nolog/NN-6880-000001/ngsSraView.do, NN-6880 https://nabic.rda.go.kr/nolog/NN-6881-000001/ngsSraView.do, NN-6881 https://nabic.rda.go.kr/nolog/NN-6882-000001/ngsSraView.do, NN-6882 https://nabic.rda.go.kr/nolog/NN-6883-000001/ngsSraView.do, NN-6883 https://nabic.rda.go.kr/nolog/NN-6884-000001/ngsSraView.do, NN-6884 https://nabic.rda.go.kr/nolog/NN-6885-000001/ngsSraView.do, NN-6885 https://nabic.rda.go.kr/nolog/NN-6886-000001/ngsSraView.do, NN-6886 https://nabic.rda.go.kr/nolog/NN-6887-000001/ngsSraView.do, NN-6887 https://nabic.rda.go.kr/nolog/NN-6888-000001/ngsSraView.do, NN-6888 https://nabic.rda.go.kr/nolog/NN-6889-000001/ngsSraView.do, NN-6889 https://nabic.rda.go.kr/nolog/NN-6891-000001/ngsSraView.do, NN-6891 https://nabic.rda.go.kr/nolog/NN-6892-000001/ngsSraView.do, NN-6892, NN-7279 https://nabic.rda.go.kr/nolog/NN-7279-000001/ngsSraView.do.

## Author contributions

J-NK: Data curation, Writing – original draft. MH: Resources, Writing – review & editing. C-KK: Investigation, Writing – review & editing. S-HY: Validation, Writing – review & editing. S-ML: Conceptualization, Supervision, Writing – review & editing.
