# Supplementary material for: Enhancing transcriptome analysis in medicinal plants: multiple unigene sets in Astragalus membranaceus
Source: Front Plant Sci. 2024 Feb 7;15:1301526. doi: 10.3389/fpls.2024.1301526 (PMC10879423; doi:10.3389/fpls.2024.1301526)
Supplement: Supplementary file 1 [file DataSheet_1.docx]

Supplementary Figures

Enhancing Transcriptome Analysis in Medicinal Plants: Multiple Unigene Sets in *Astragalus membranaceus*

Ji-Nam Kang, Mok Hur, Chang-Kug Kim, So-Hee Yang, Si-Myung Lee

# Supplementary Figures

## Supplementary Figures


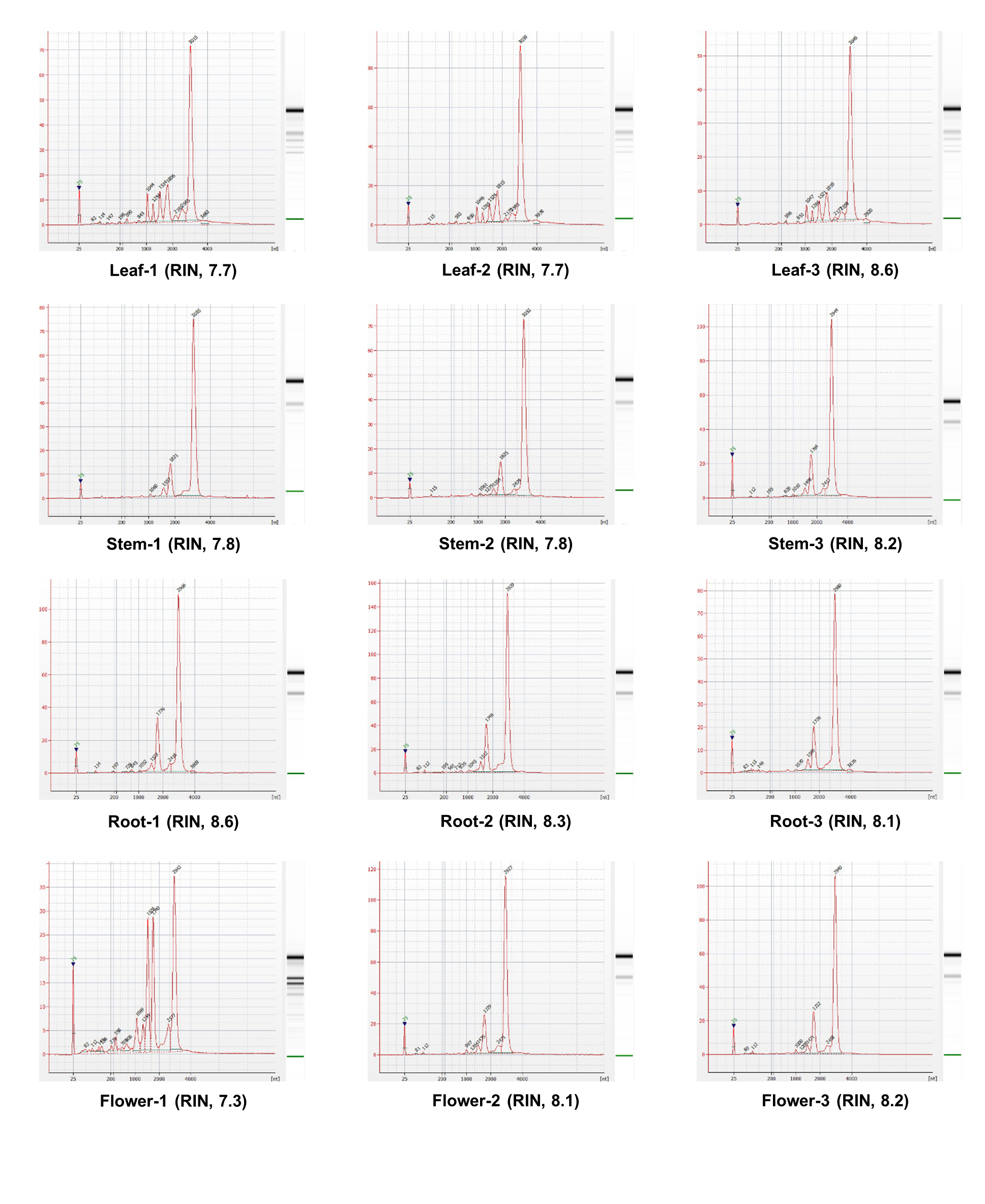


**Supplementary Figure 1. RNA integrity number (RIN) values for 12 RNA samples.**


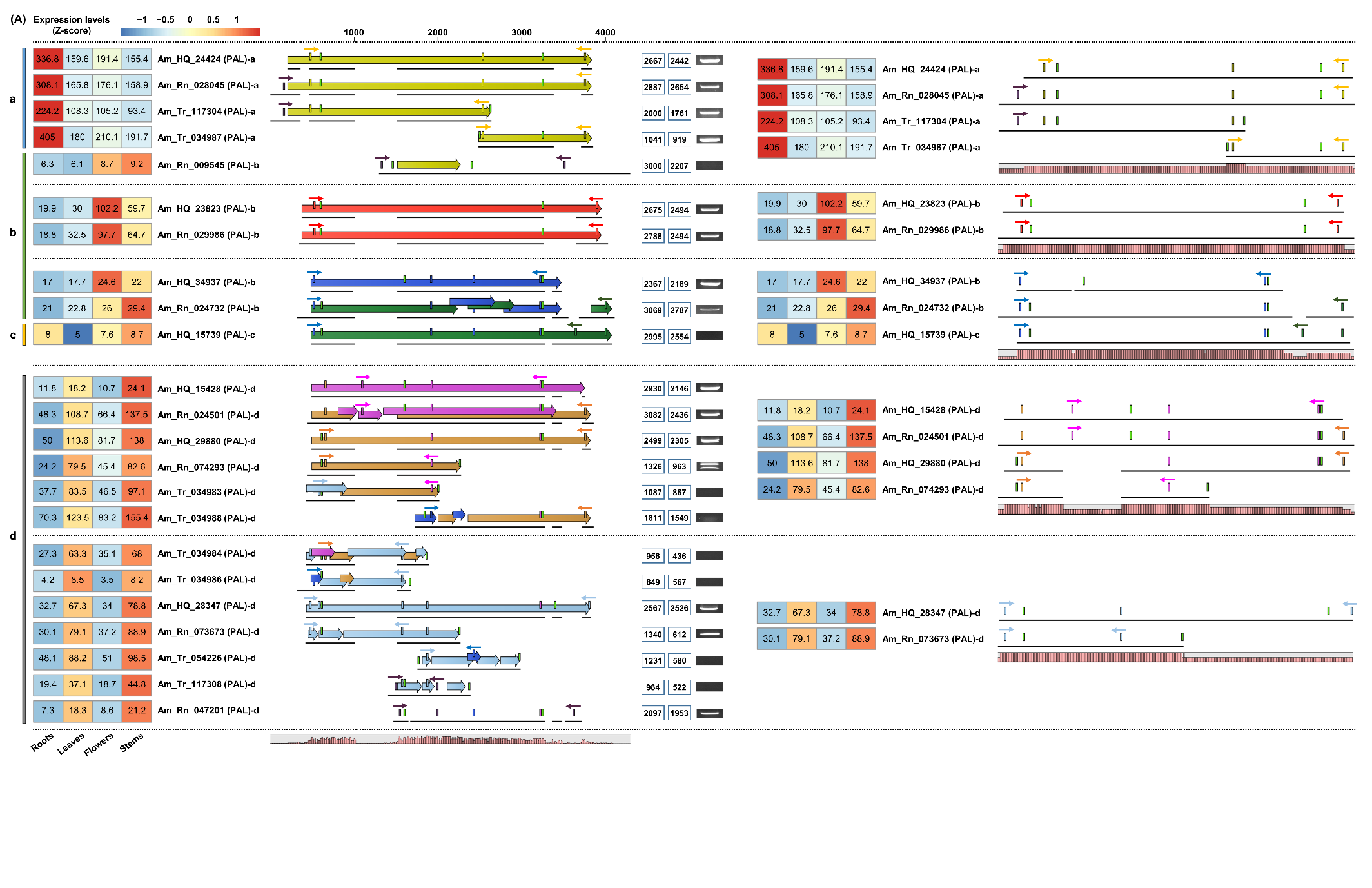


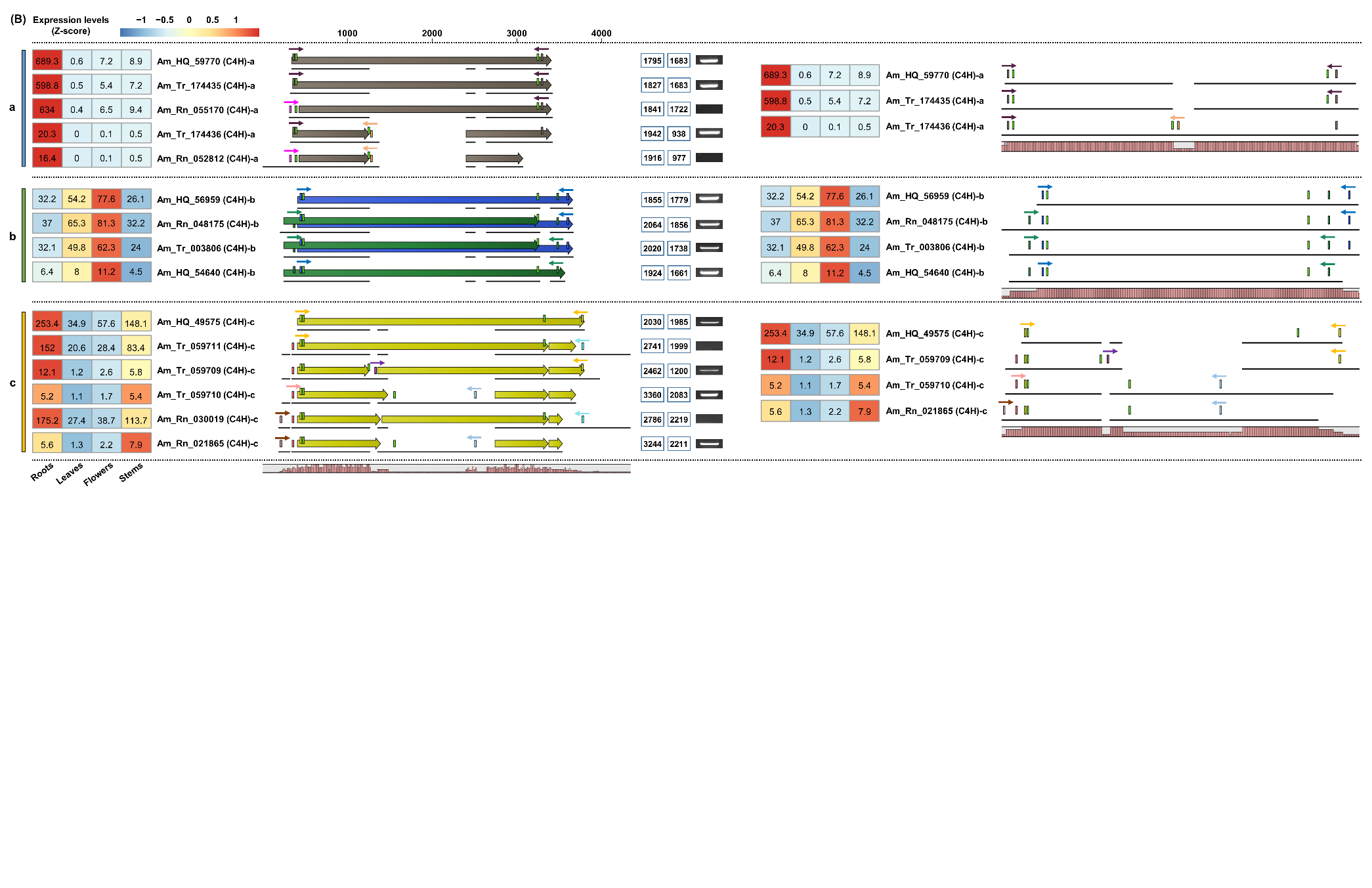


**
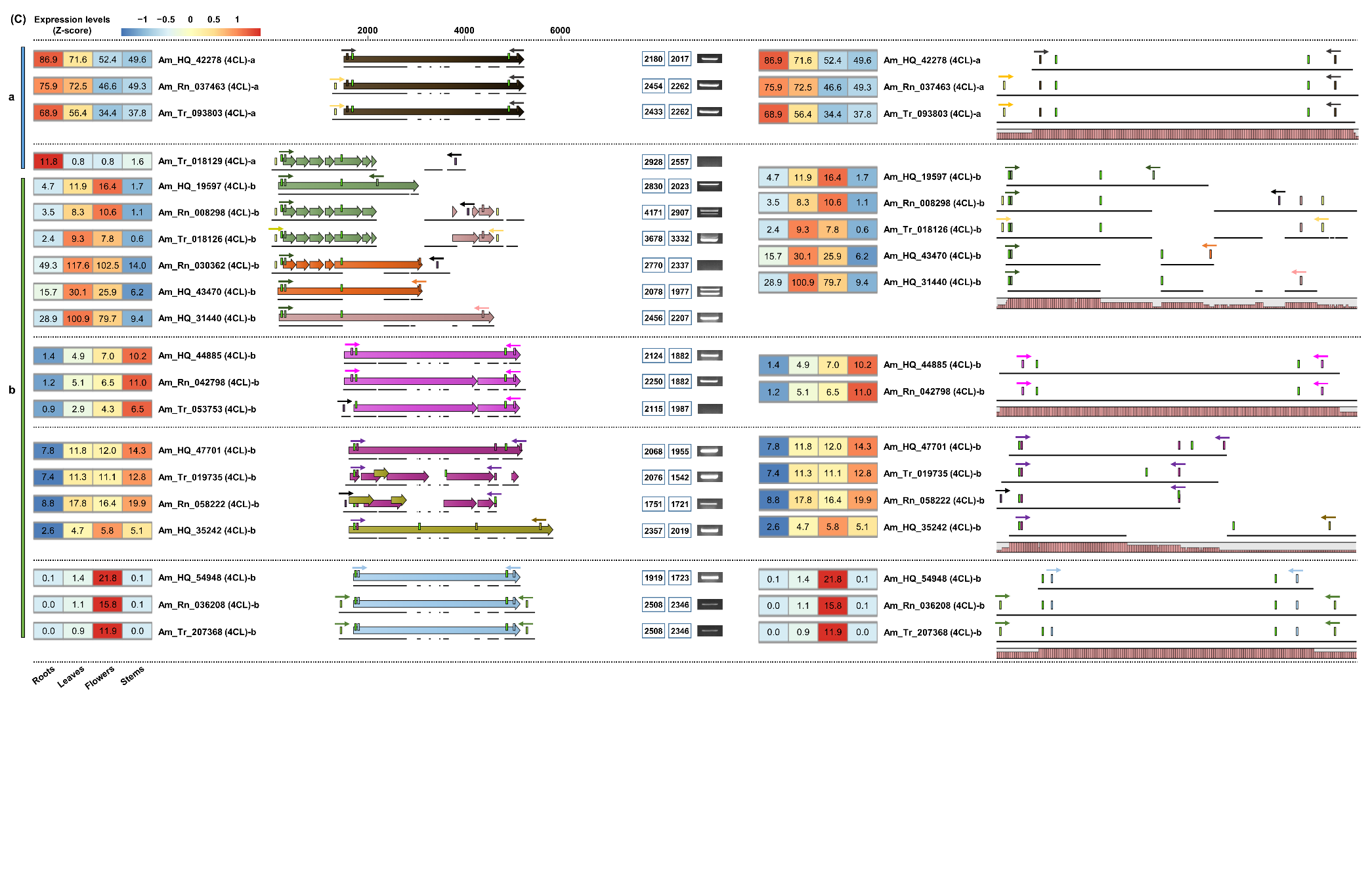
**

**
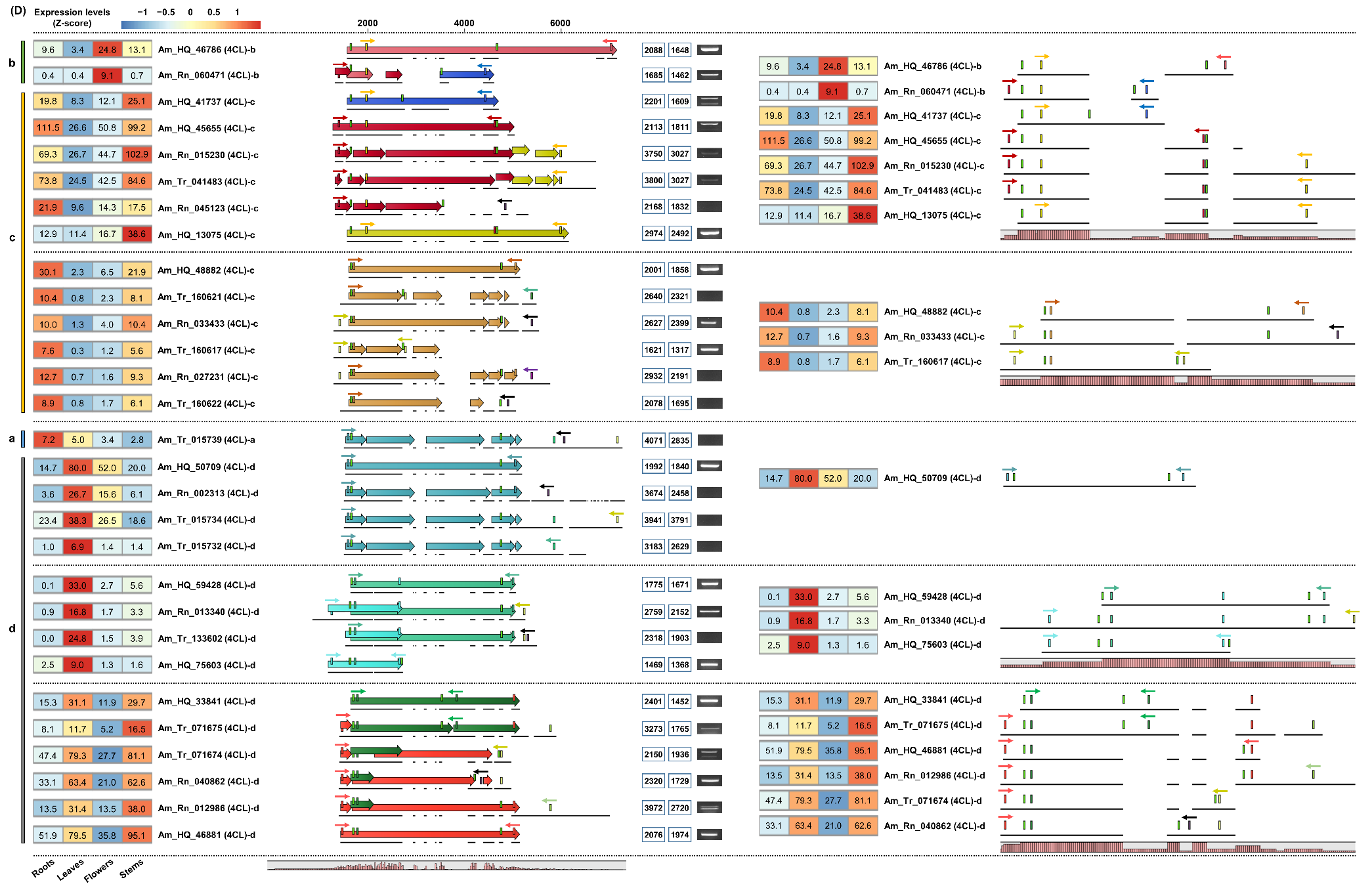
**

**
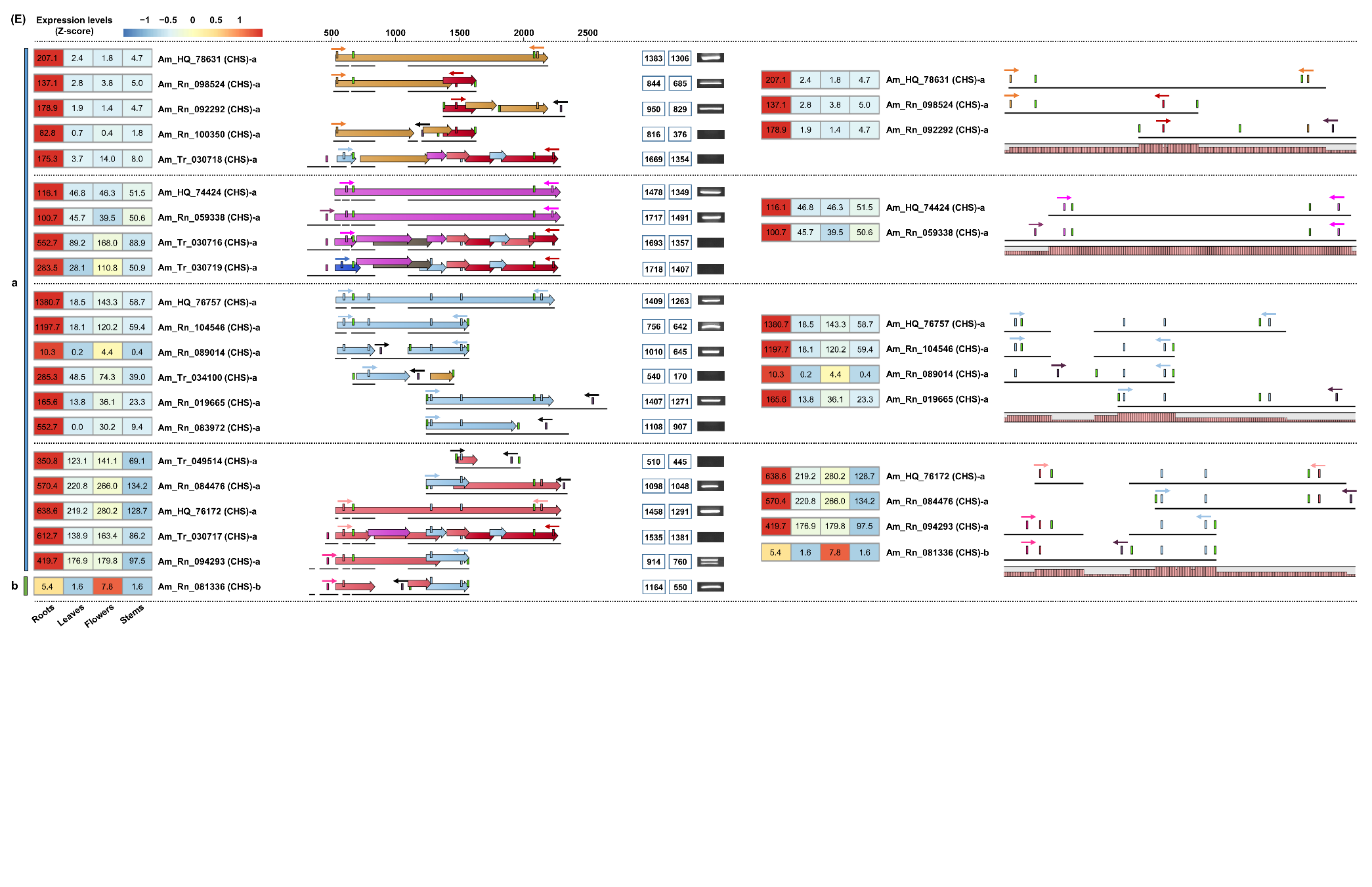
**

**
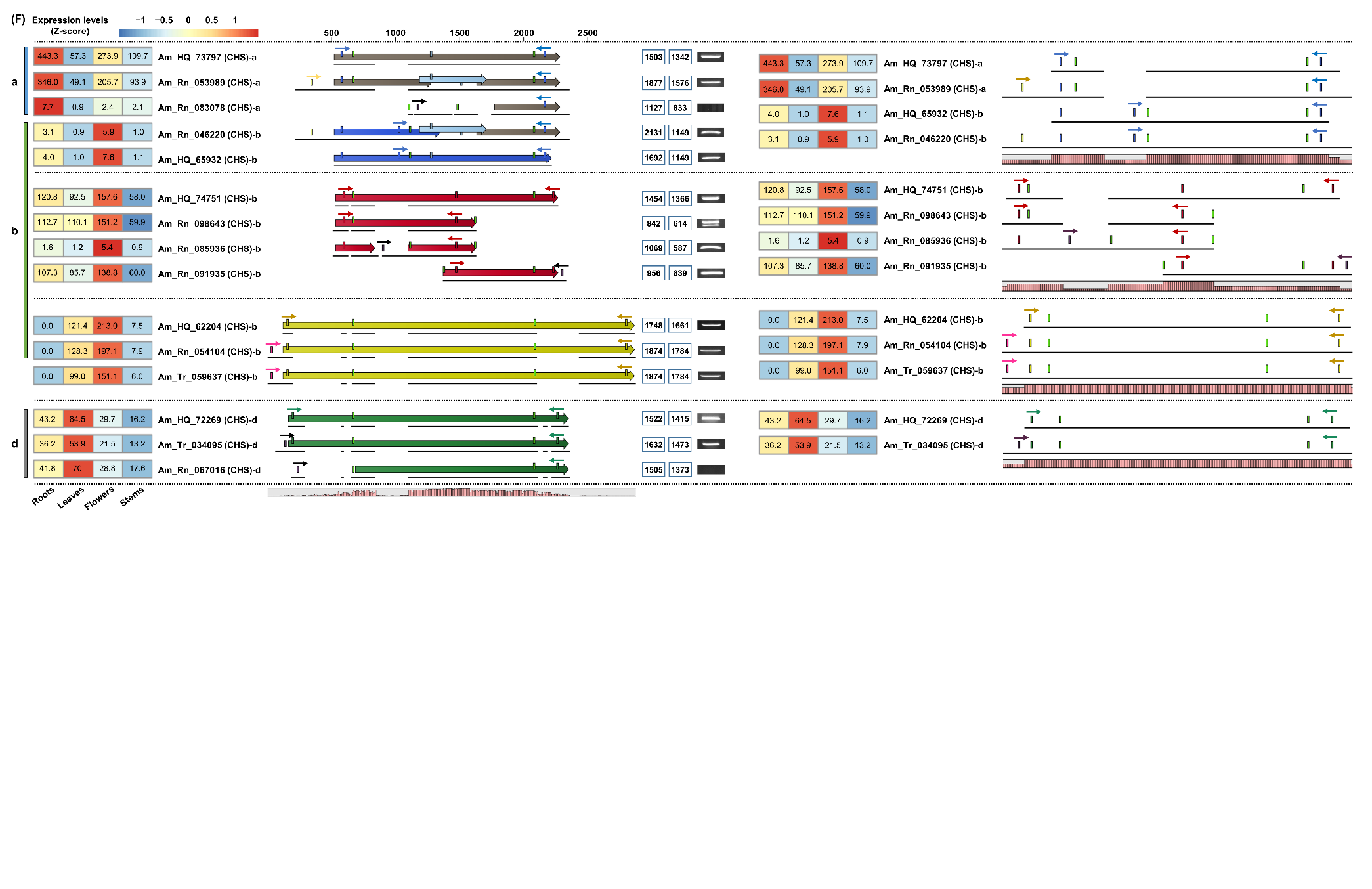
**

**
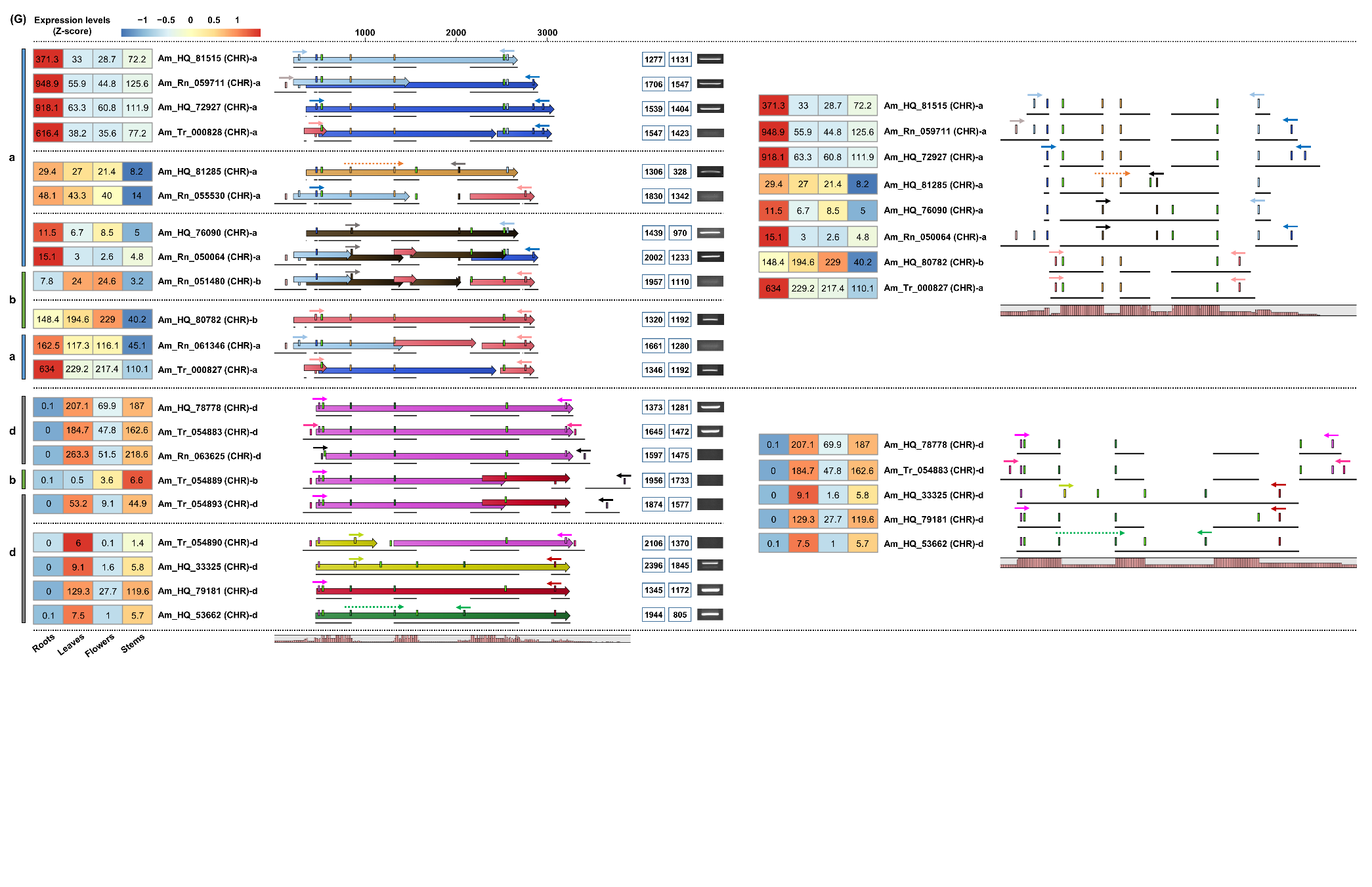
**

**
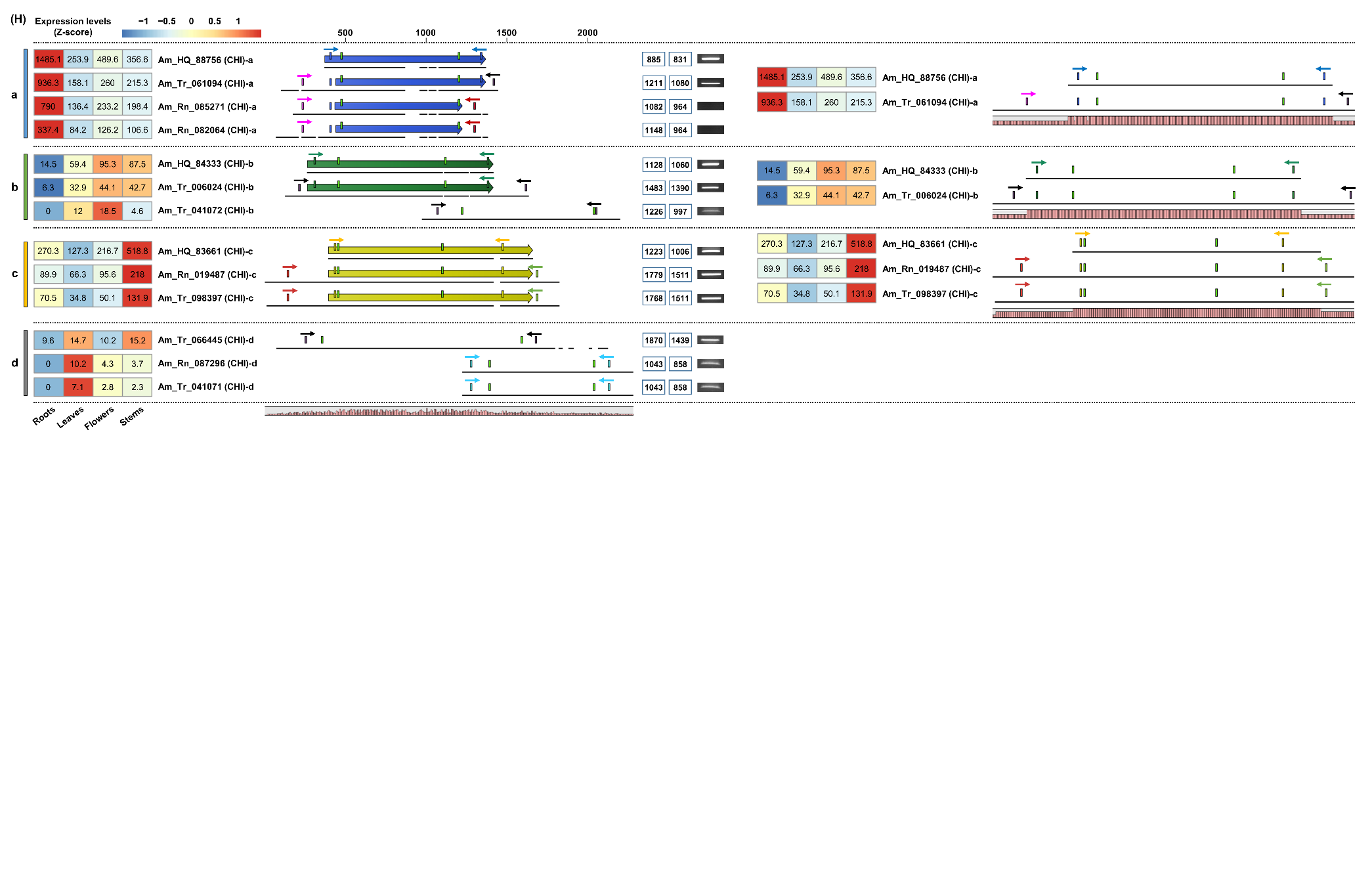
**

**
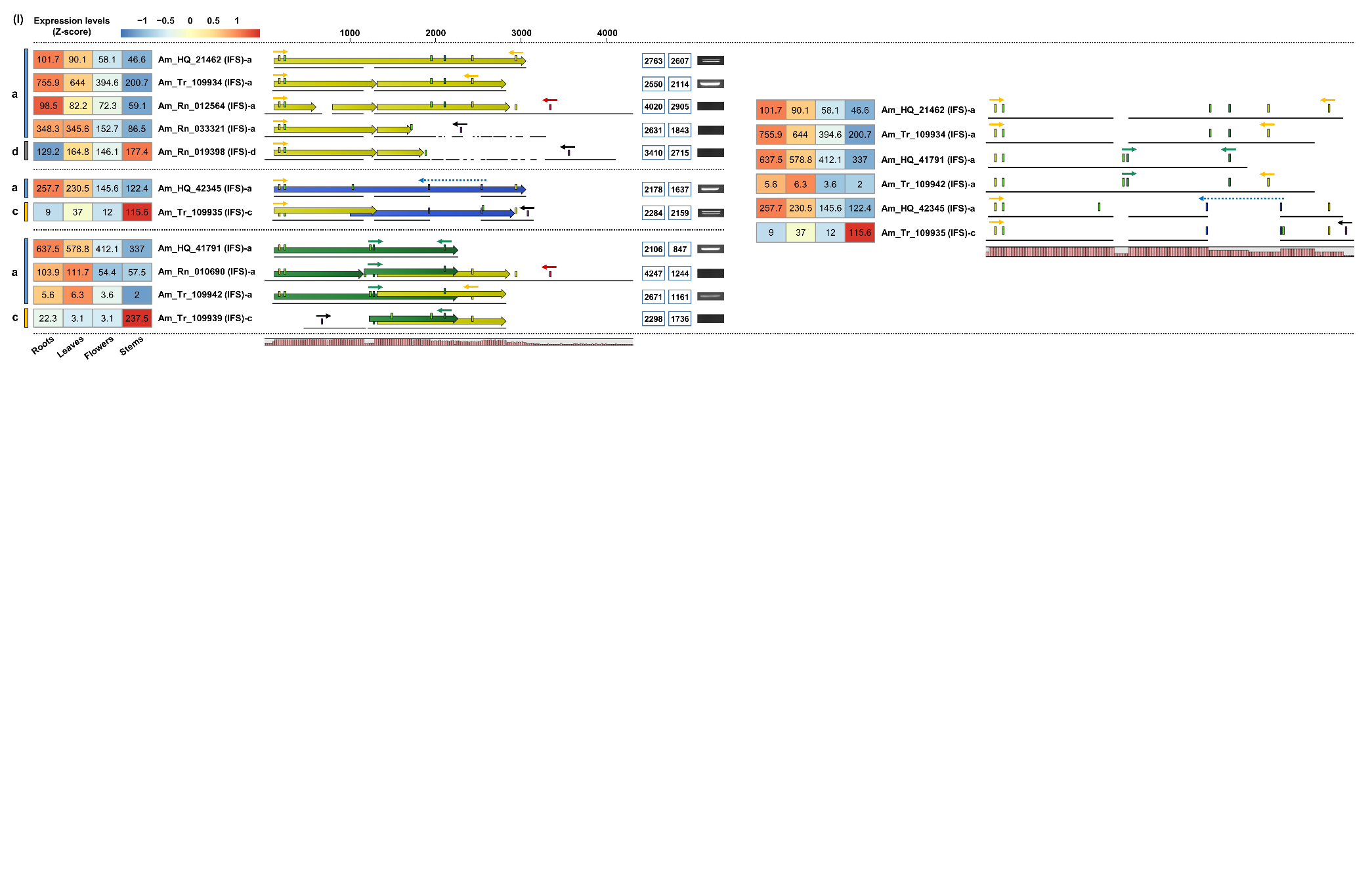
**

**
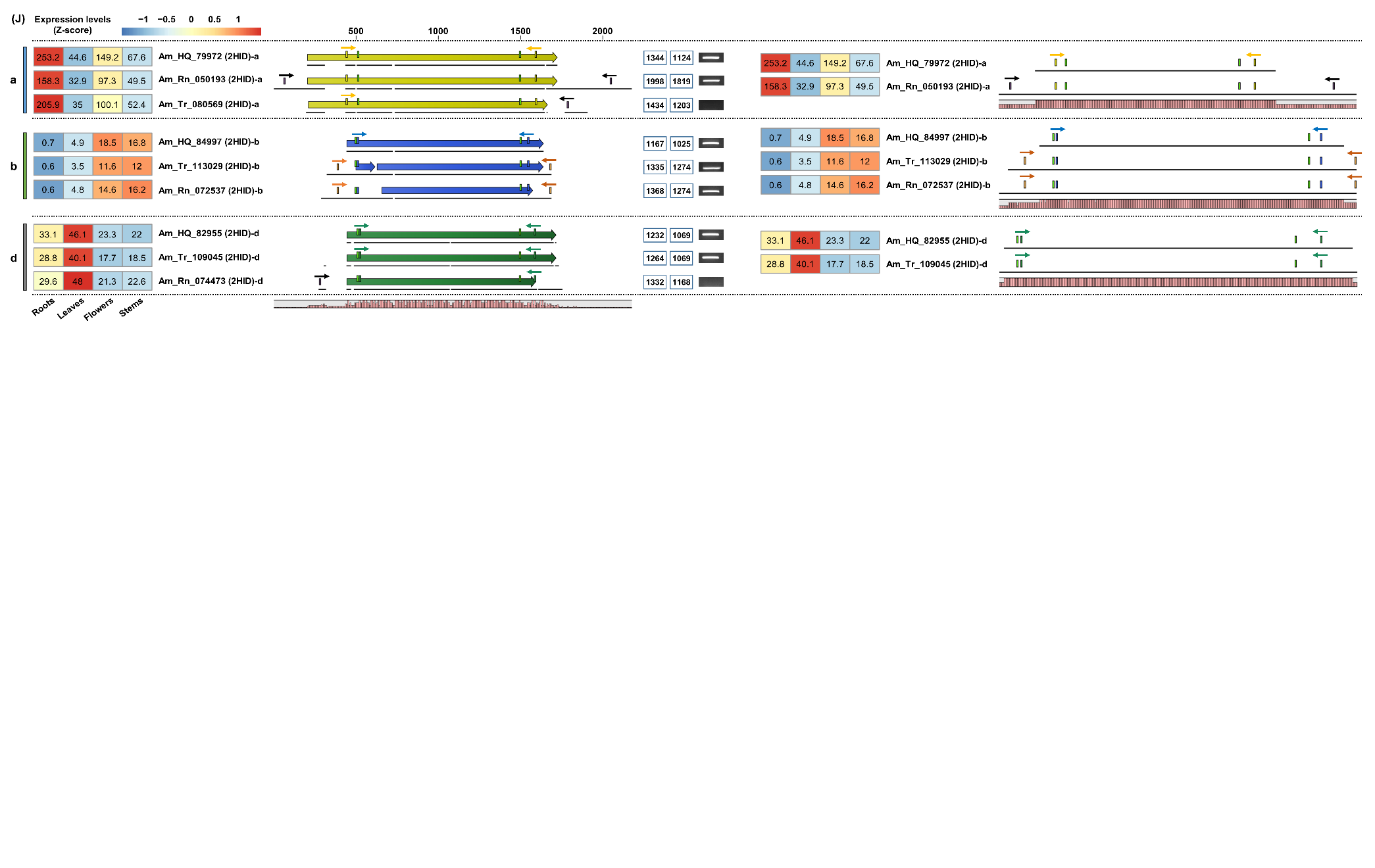
**

**
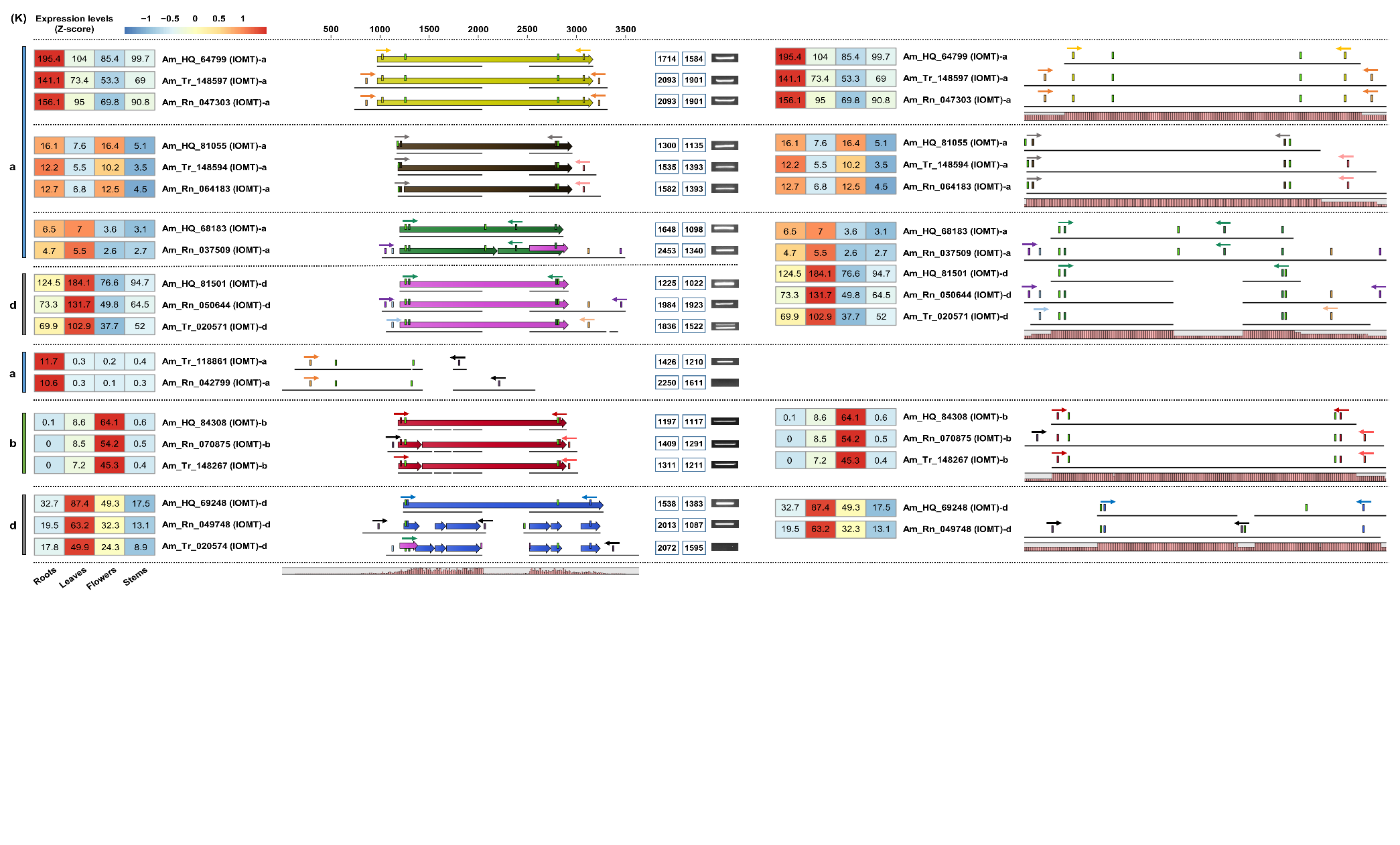
**

**
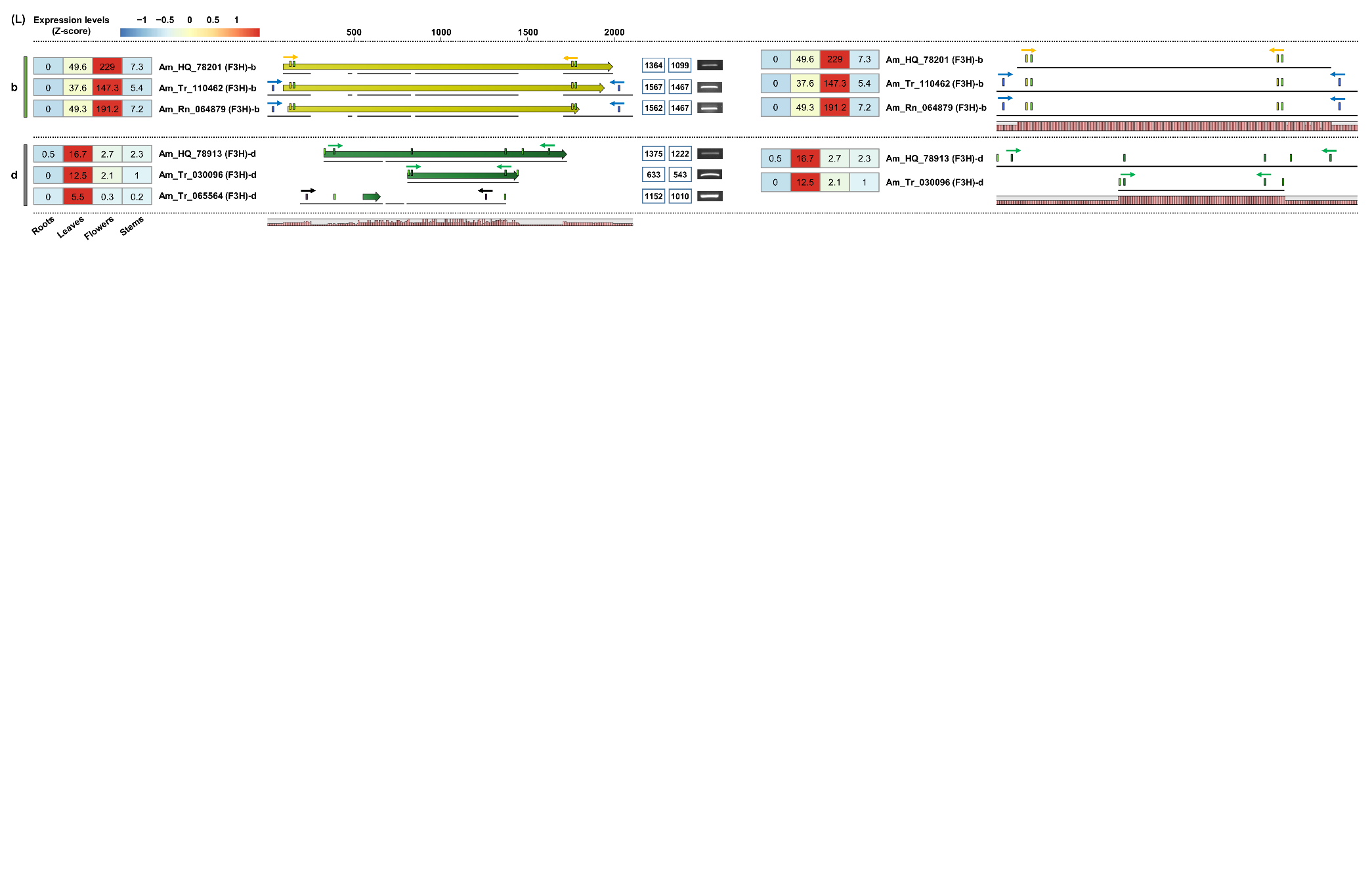
**

**
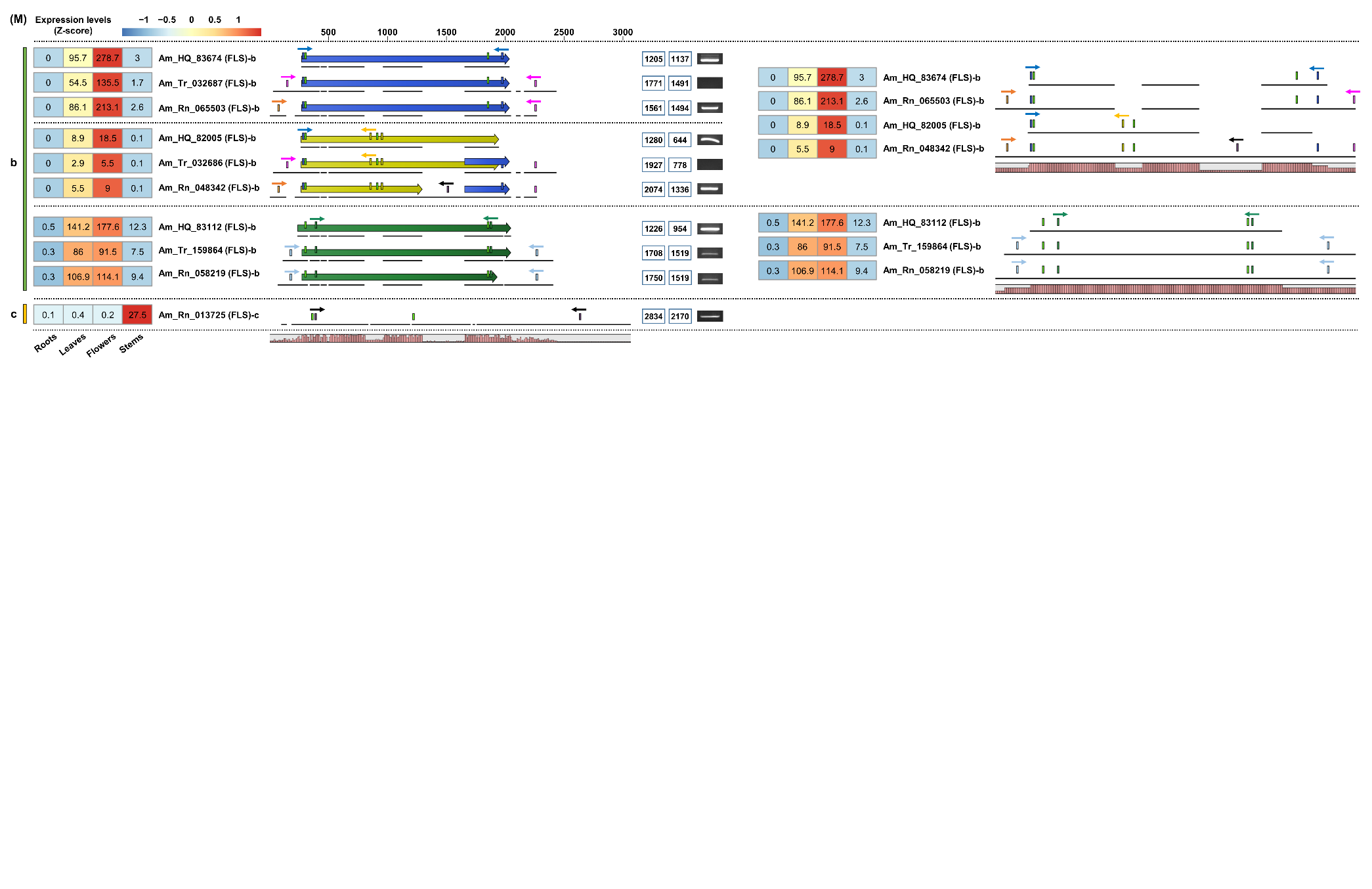
**

**
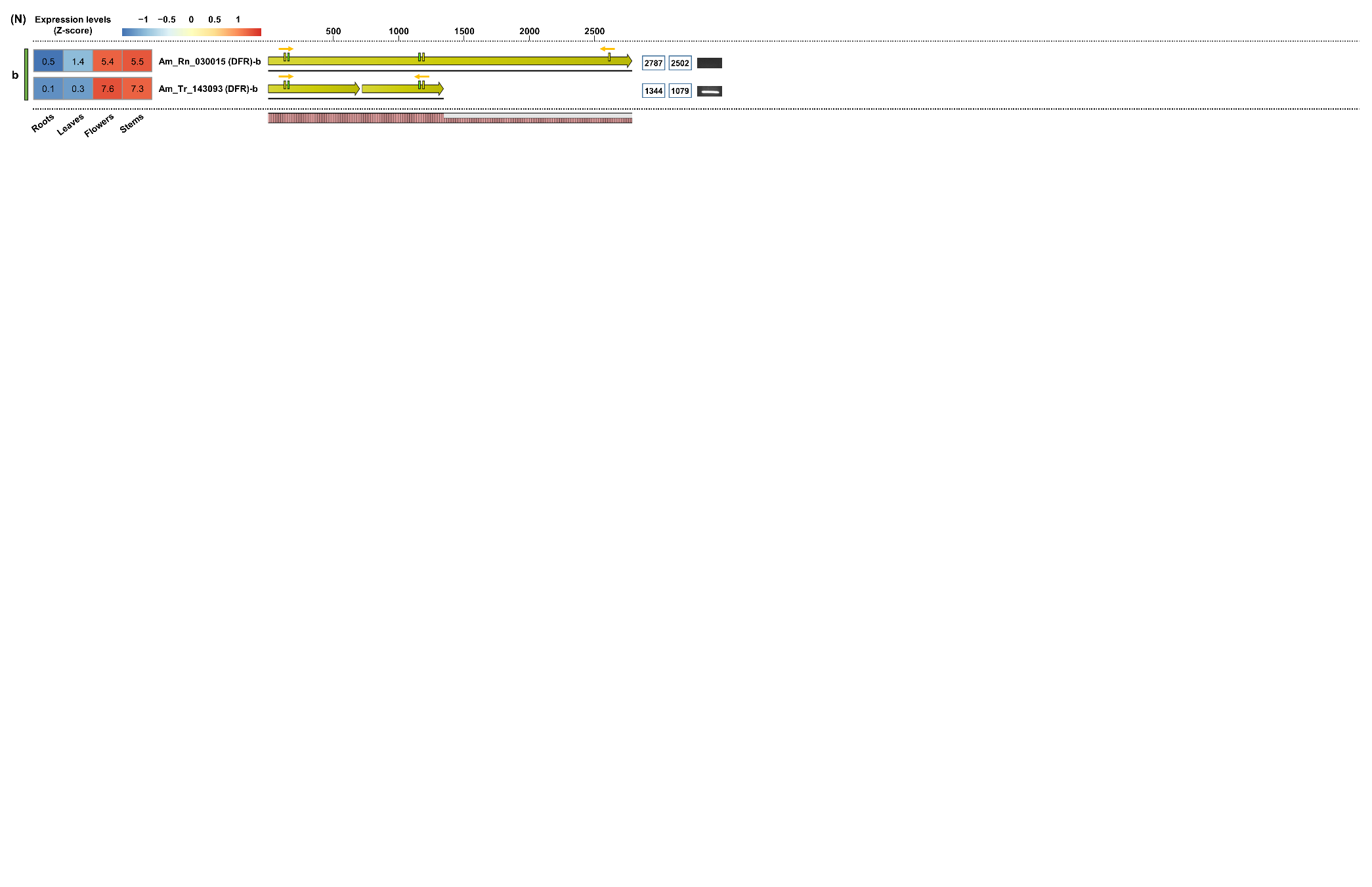
**

**
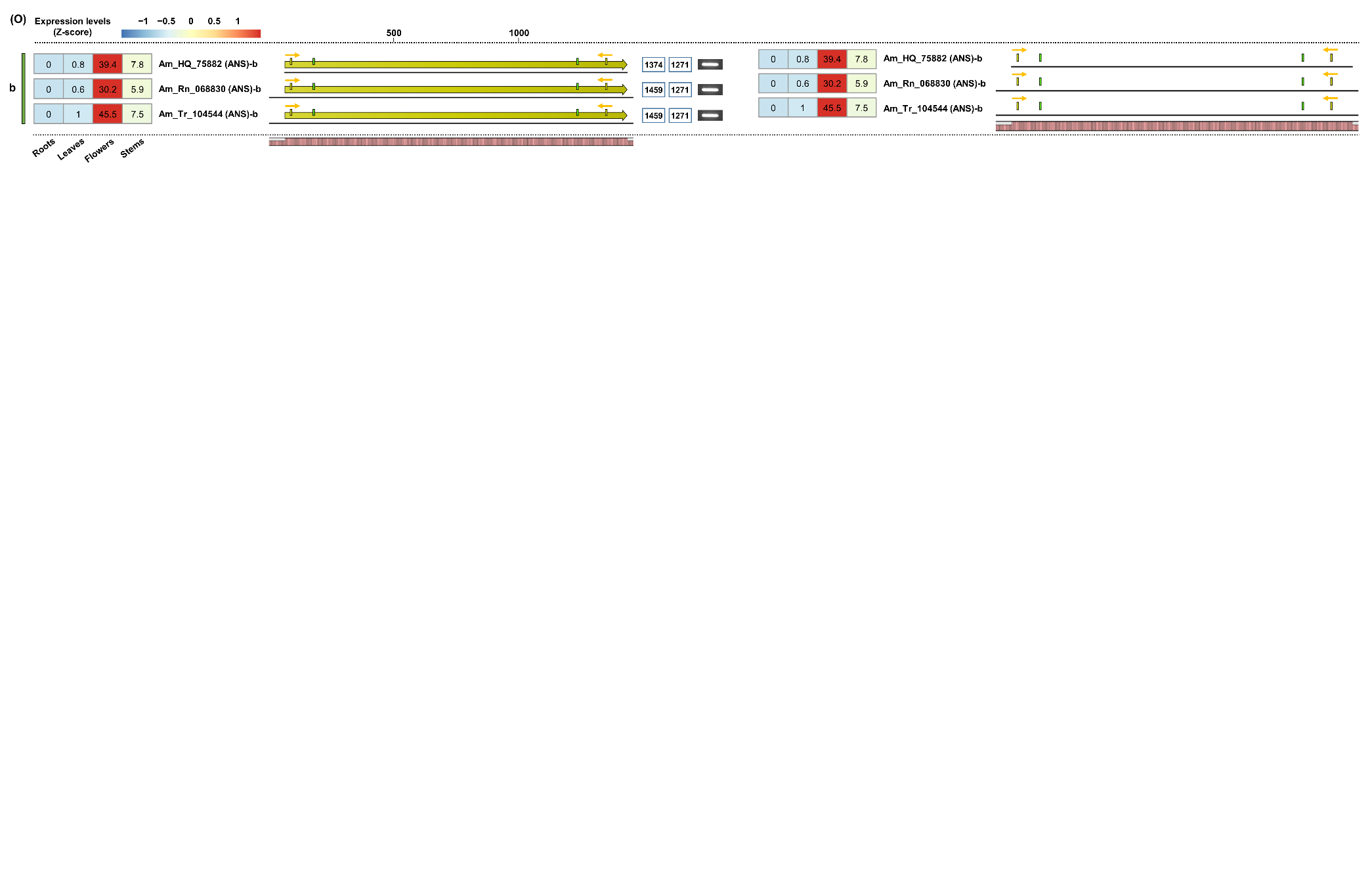
**

**Supplementary Figure 2. Schematic diagram for structural comparison of phenylpropanoid biosynthesis-related unigenes derived from three unigene sets.** The structures of unigenes encoding the key enzymes PAL (**A**), C4H (**B**), 4CL (**C and D**), CHS (**E and F**), CHR (**G**), CHI (**H**), IFS (**I**), 2HID (**J**), IOMT (**K**), F3H (**L**), FLS (**M**), DFR (**N**), and ANS (**O**) involved in phenylpropanoid biosynthesis were compared using alignment, and specific primers were prepared for RT-PCR verification. In the structural diagram, the range within the light green box represented the predicted CDS region. Homologous sequence regions are indicated by identically colored boxed arrows. Colored boxes indicate specific primer positions. The numbers in the blue line box represent the total length and amplification size of the unigenes, respectively. Homology graphs were displayed at the bottom of the schematic diagram using a bar plot (right red bars).

**
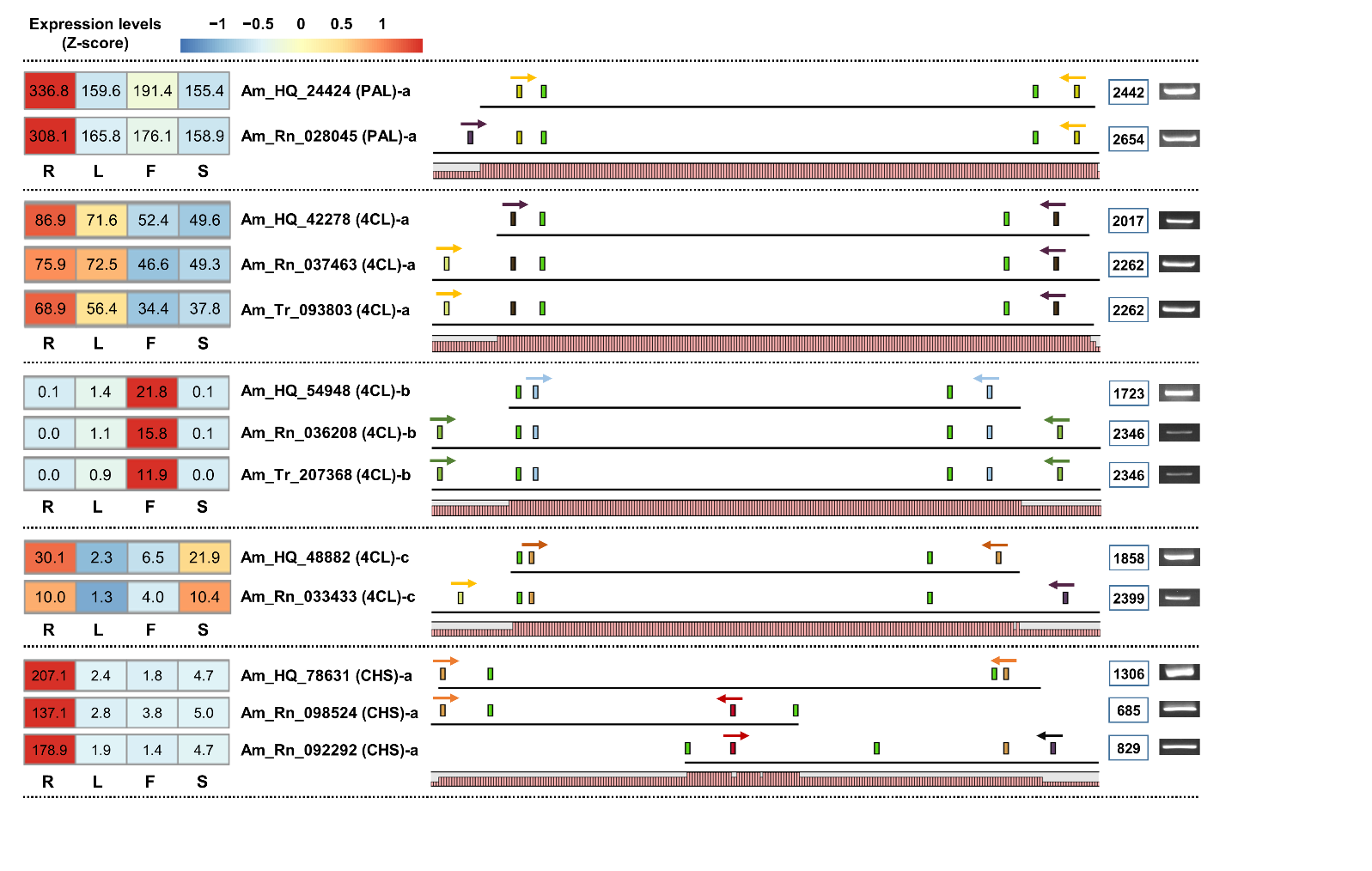
**

**
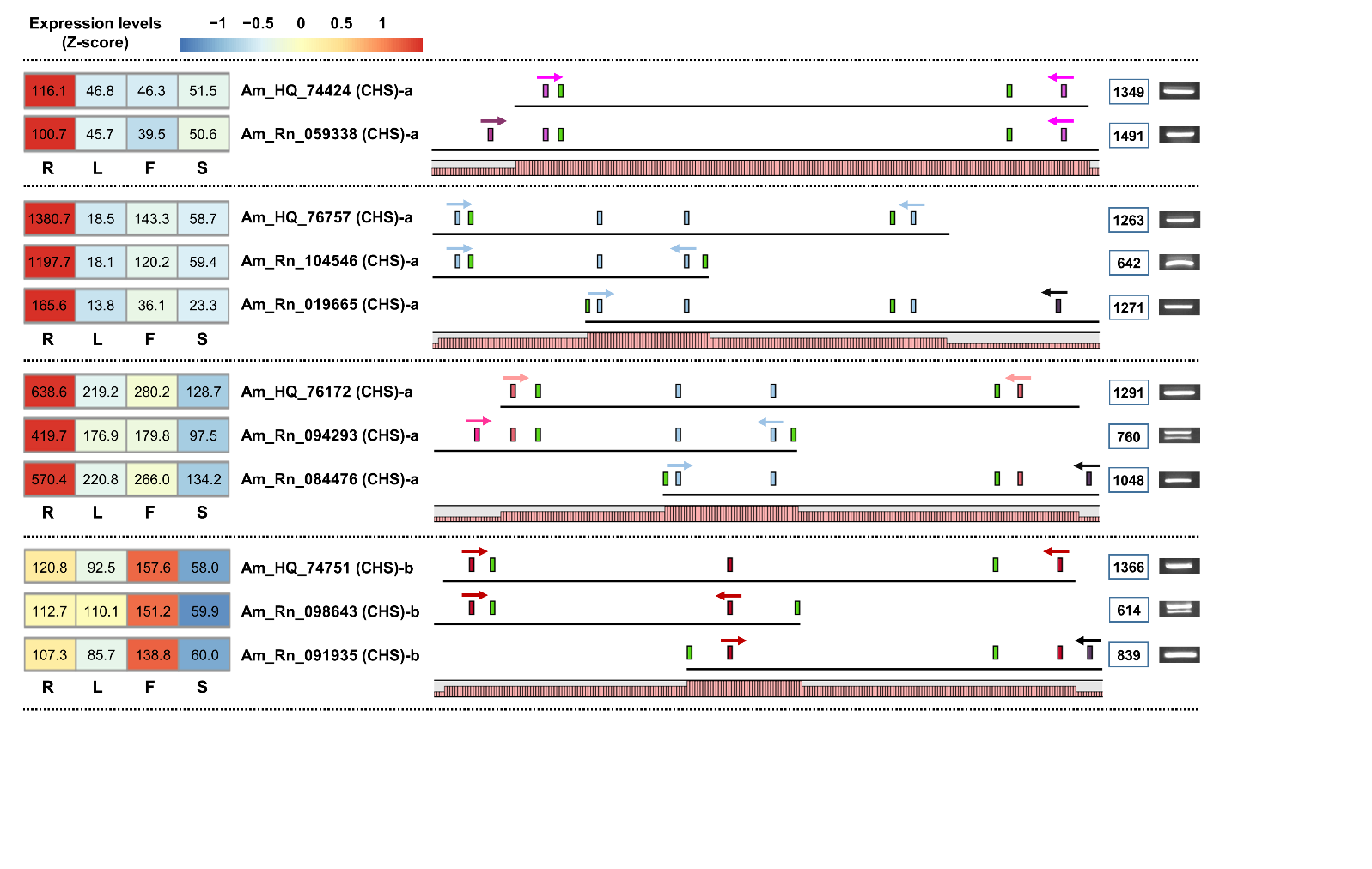
**

**
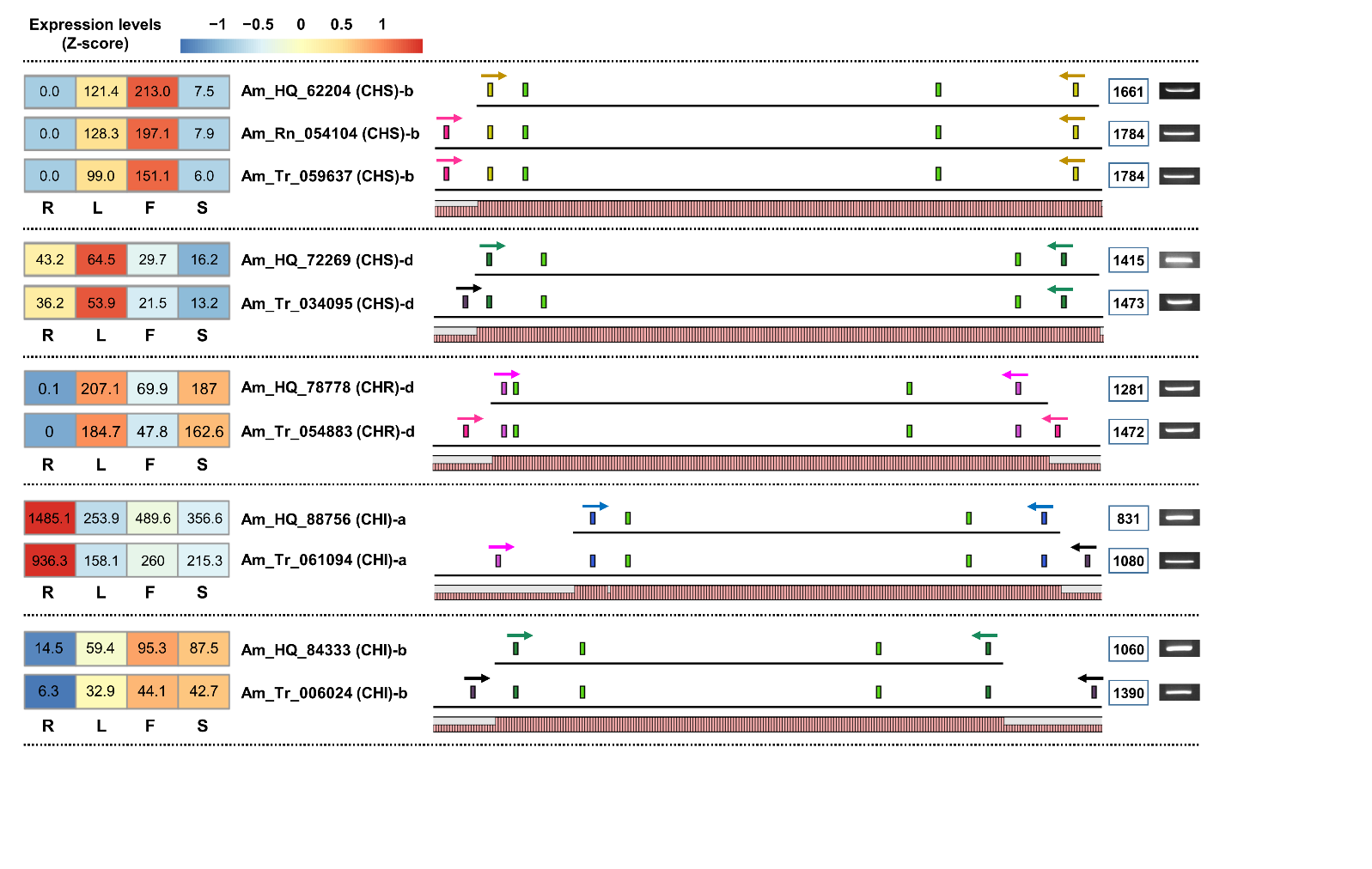

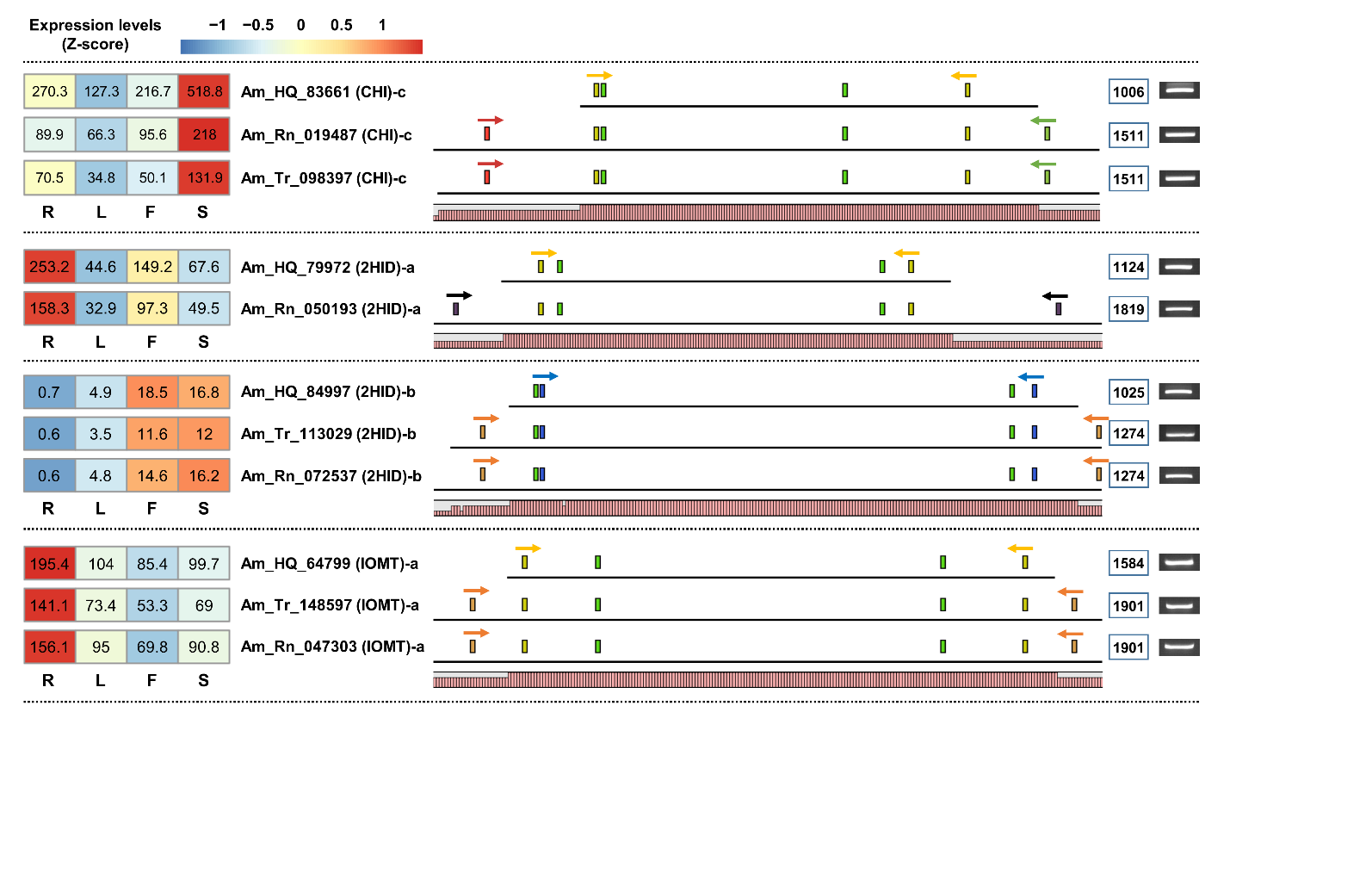
**

**
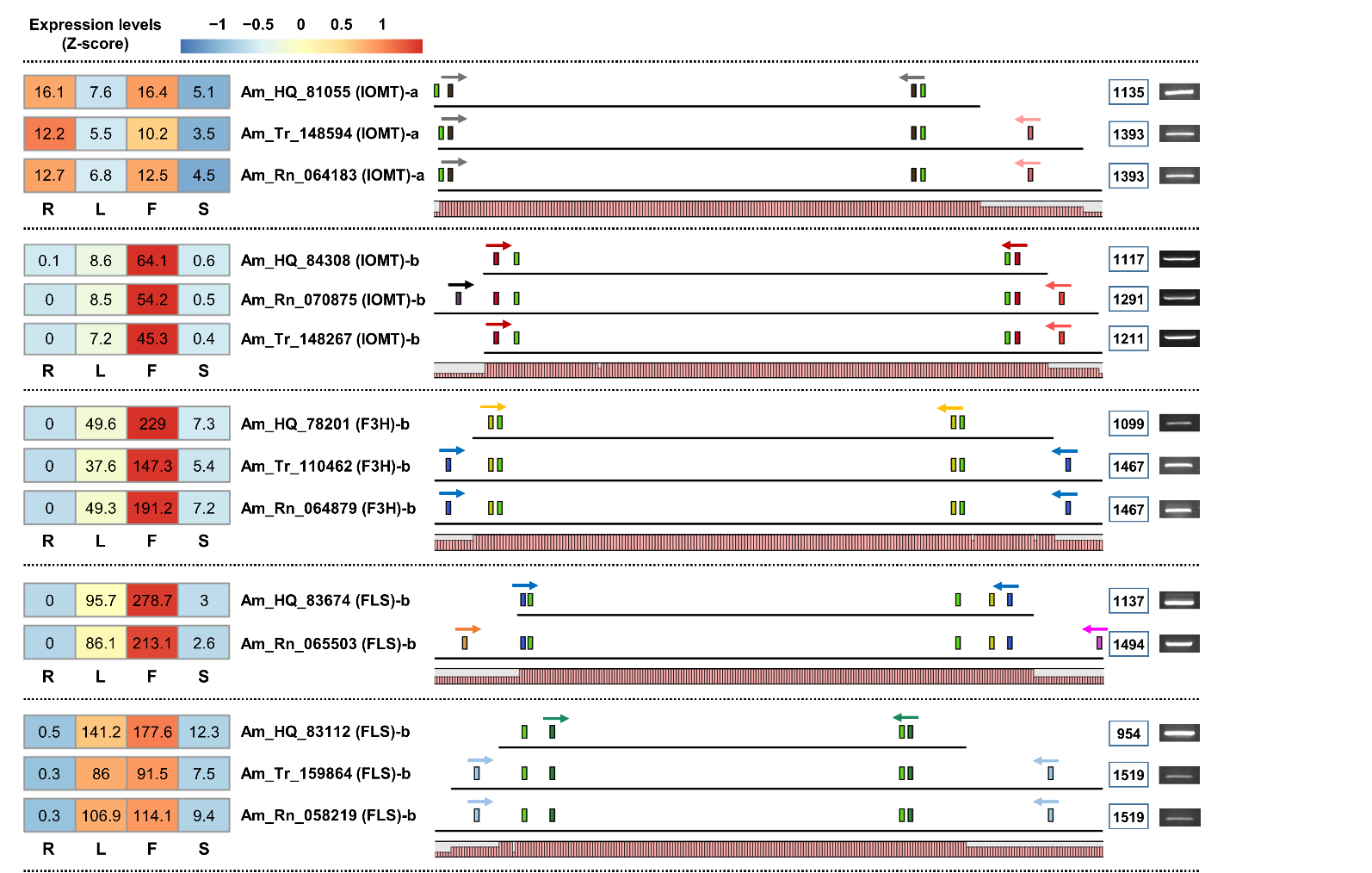
**

**
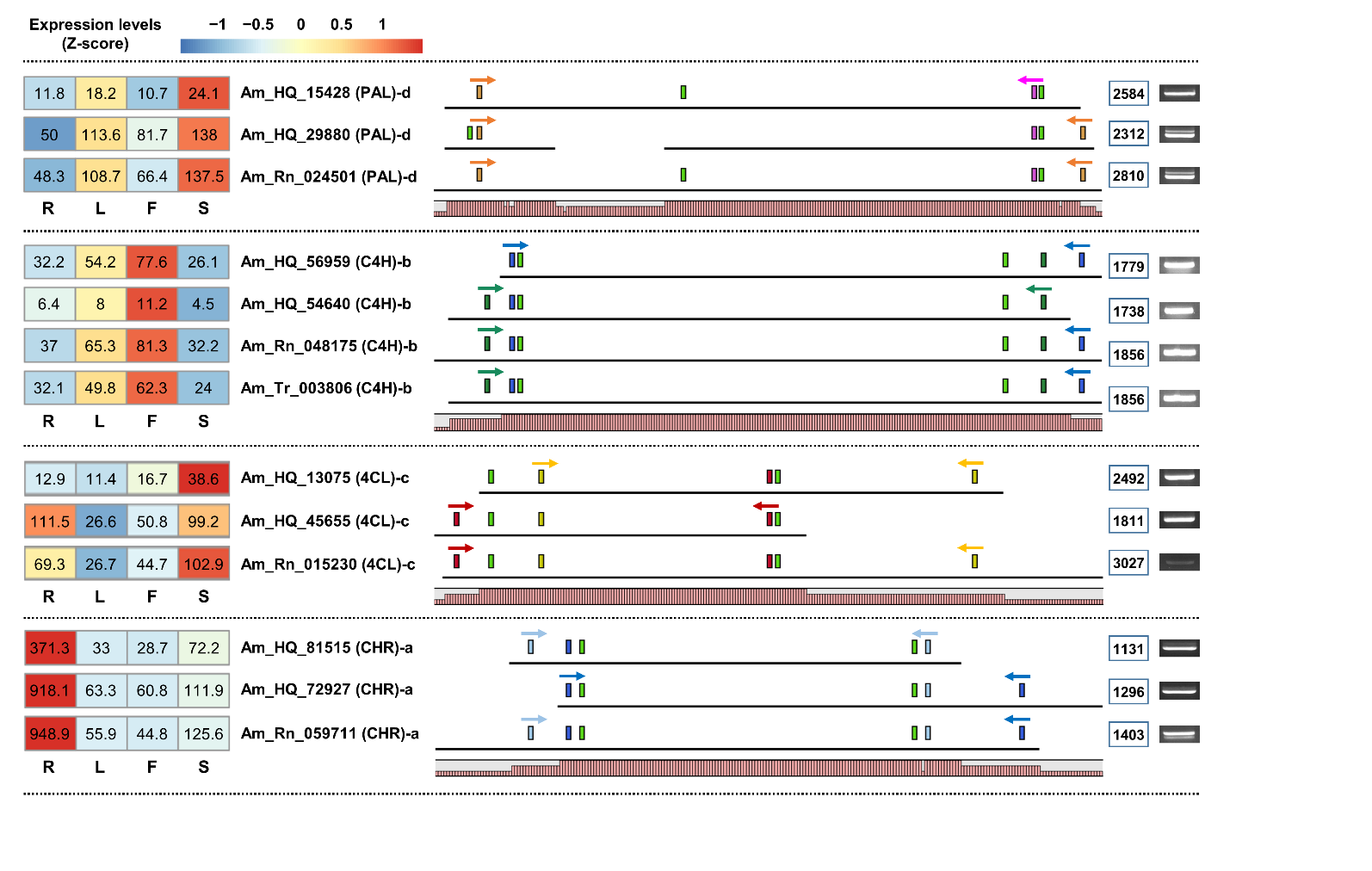
Supplementary Figure 3. Incomplete UTR termini of phenylpropanoid-related unigenes derived from Set-1.** The range within the light green box represents the predicted CDS region. Colored boxes indicate specific primer positions. The numbers in the blue line box represent the amplification size of the unigenes. Homology graphs are displayed at the bottom of the schematic diagram using a bar plot (right red bars).

**
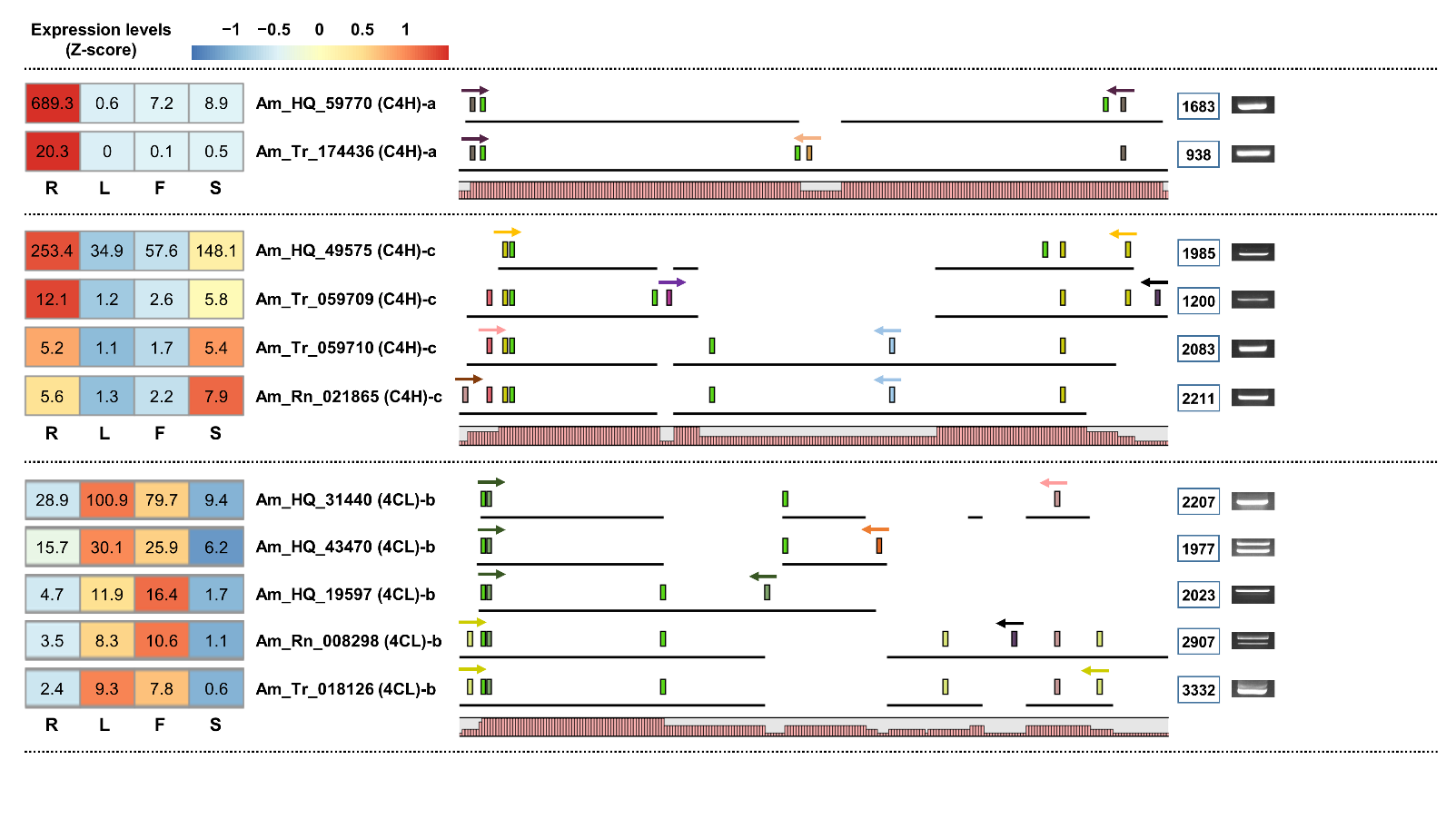
**

**
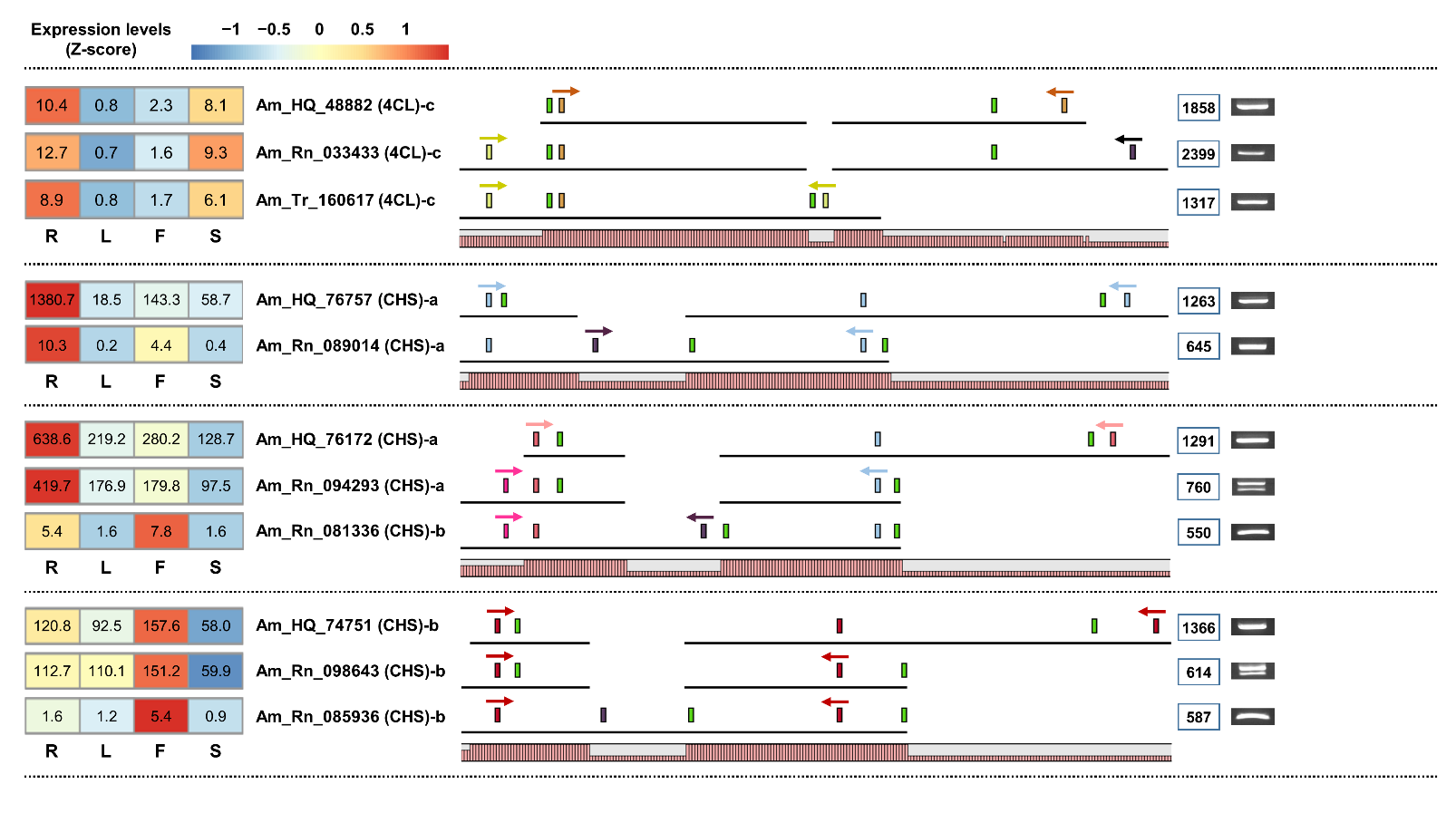
**

**
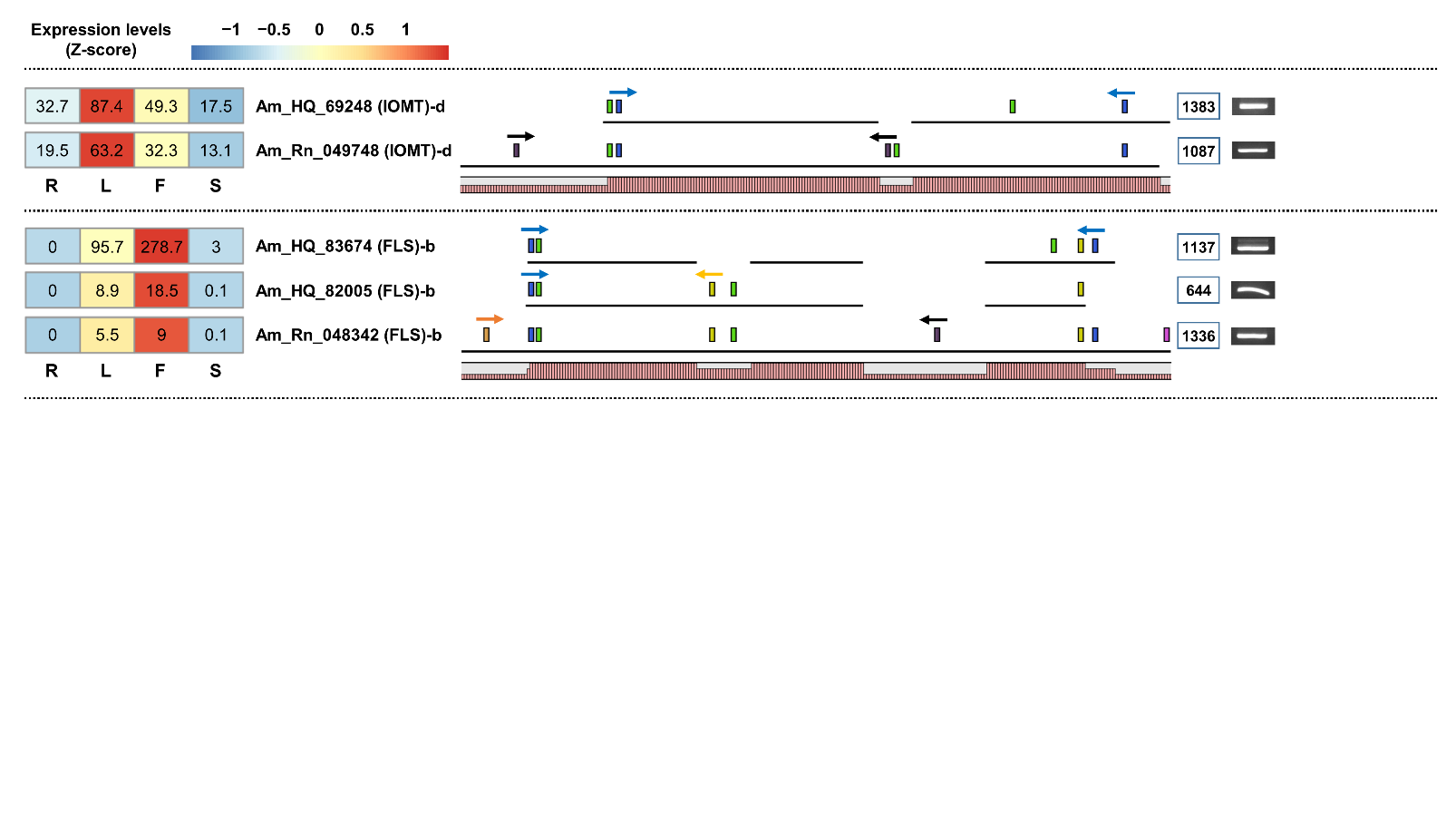
**

**Supplementary Figure 4. Schematic diagram of different isoform types detected from set-2 and set-3.** The range within the light green box represents the predicted CDS region. Colored boxes indicate specific primer positions. The numbers in the blue line box represent the amplification size of the unigenes. Homology graphs are displayed at the bottom of the schematic diagram using a bar plot (right red bars).

**
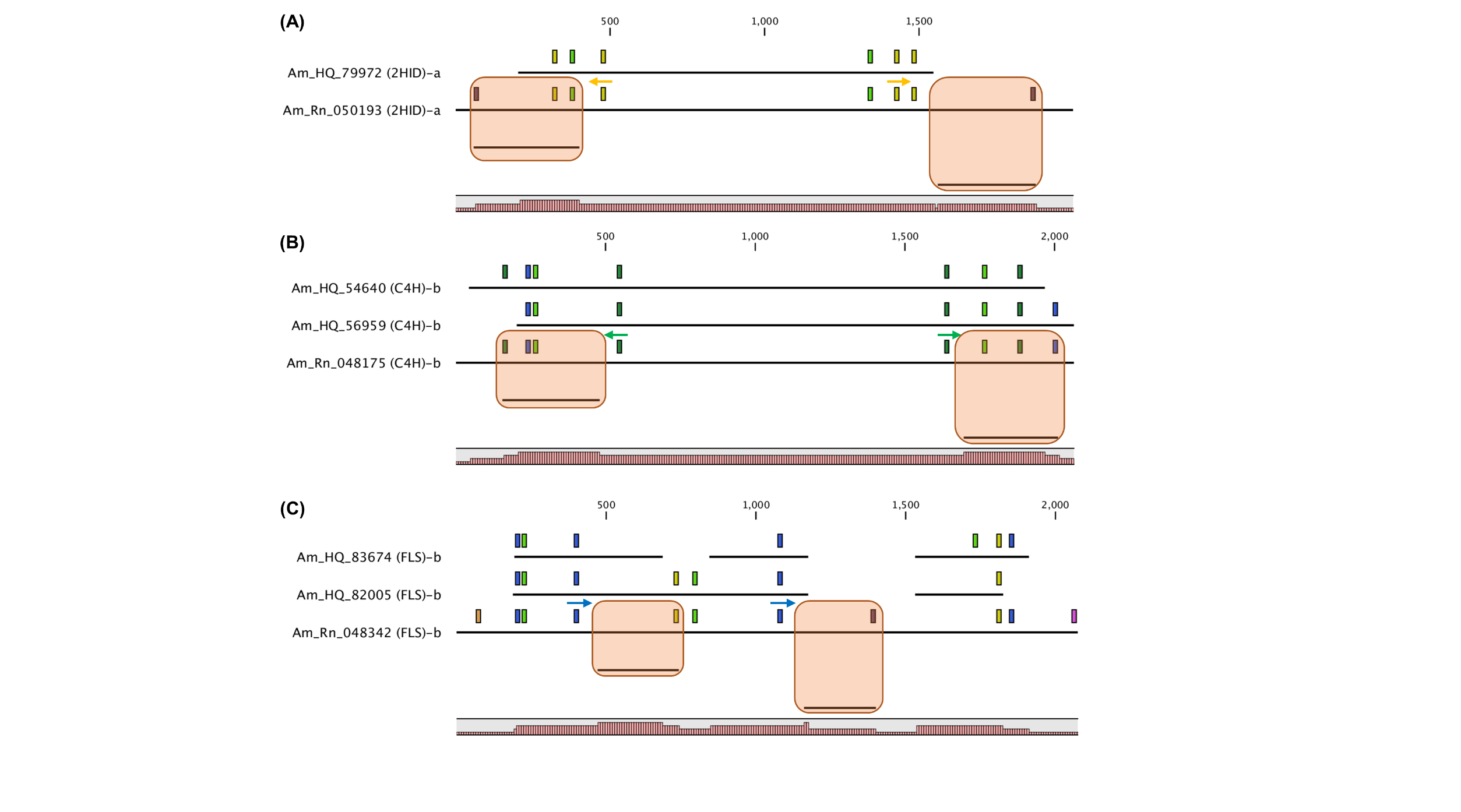
**

**Supplementary Figure 5. Actual sequence verification using direct sequencing.** Partial sequences (orange boxes) of 2HID (**A**), C4H (**B**), and FLS (**C**) encoded unigenes derived from set-2 were confirmed using direct sequencing. The range within the light green box represents the predicted CDS region. Arrows indicate primer positions used for direct sequencing. Homology graphs are displayed at the bottom of the schematic diagram using a bar plot (right red bars).
